# Suppression of the Arboviruses Dengue and Chikungunya Using a Dual-Acting Group-I Intron Coupled with Conditional Expression of the Bax C-Terminal Domain

**DOI:** 10.1371/journal.pone.0139899

**Published:** 2015-11-18

**Authors:** James R. Carter, Samantha Taylor, Tresa S. Fraser, Cheryl A. Kucharski, James L. Dawson, Malcolm J. Fraser

**Affiliations:** Department of Biological Sciences, Eck Institute of Global Health, University of Notre Dame, Notre Dame, Indiana, United States of America; Virginia Tech, UNITED STATES

## Abstract

In portions of South Asia, vectors and patients co-infected with dengue (DENV) and chikungunya (CHIKV) are on the rise, with the potential for this occurrence in other regions of the world, for example the United States. Therefore, we engineered an antiviral approach that suppresses the replication of both arboviruses in mosquito cells using a single antiviral group I intron. We devised unique configurations of internal, external, and guide sequences that permit homologous recognition and splicing with conserved target sequences in the genomes of both viruses using a single trans-splicing Group I intron, and examined their effectiveness to suppress infections of DENV and CHIKV in mosquito cells when coupled with a proapoptotic 3' exon, ΔN Bax. RT-PCR demonstrated the utility of these introns in trans-splicing the ΔN Bax sequence downstream of either the DENV or CHIKV target site in transformed *Aedes albopictus* C6/36 cells, independent of the order in which the virus specific targeting sequences were inserted into the construct. This trans-splicing reaction forms DENV or CHIKV ΔN Bax RNA fusions that led to apoptotic cell death as evidenced by annexin V staining, caspase, and DNA fragmentation assays. TCID50-IFA analyses demonstrate effective suppression of DENV and CHIKV infections by our anti-arbovirus group I intron approach. This represents the first report of a dual-acting Group I intron, and demonstrates that we can target DENV and CHIKV RNAs in a sequence specific manner with a single, uniquely configured CHIKV/DENV dual targeting group I intron, leading to replication suppression of both arboviruses, and thus providing a promising single antiviral for the transgenic suppression of multiple arboviruses.

## Introduction

The WHO estimates hundreds of millions of infections and tens of thousands of deaths each year are attributed to mosquito-borne virus related diseases, with well over half the world’s population remaining at risk for infection [1–7]. Individual outbreaks along with instances of co-infections of dengue (DENV) and chikungunya viruses (CHIKV), are on the rise due to the presence of both pathogens in a shared mosquito vector, *Aedes albopictus* [2,8–12].

Infection with one of four orthologous, but antigenically distinct DENV serotypes (designated DENV 1 through 4) can result in dengue fever (DF) or dengue hemorrhagic fever (DHF) [1]. DF and DHF are endemic to tropical and subtropical regions of the world, but global changes in climate, rapid dispersal of virus due to world travel, and migration of humans to non-tropical regions has resulted in epidemic DENV outbreaks in areas that are non-endemic for these viruses [13,14]. There are currently no consistently effective preventive control measures or approved tetravalent vaccines to combat DENV.

CHIKV is an emerging pathogen that infects humans with the principle mosquito vectors being *Ae*. *aegypti*, *Ae*. *albopictus*, and *Ae*. *vigilax* [15], the same vectors responsible for dengue virus spread [16–18]. Following a 2–12 day incubation period, clinical symptoms develop that are similar to dengue fever including high fever, a prominent rash on the thorax and face, headache, back pain, and myalgia. An intense arthralgia distinguishes CHIK fever from DF. Hemorrhagic fever resulting from CHIKV infection, has been reported during outbreaks in Thailand [2].

CHIKV has been transmitted throughout Asia and Africa since the initial discovery of this virus in Tanzania in 1952 [2,19–25]. Importation of this virus into Europe and the USA resulted from infected travelers returning from endemic areas with high incidences of CHIKV infection and *Ae*. *albopictus* transmission [26], underscoring the potential for a worldwide CHIKV epidemic and the need for novel therapies to effectively combat the spread of this virus. Most recently CHIKV transmission has occurred in the French Riviera [27], the Caribbean islands, and the United States [28–30].

Our lab has been surveying ribozymes as suppressive agents against arbovirus infection for potential use in generating refractory transgenic mosquitoes. We previously examined the effectiveness of hammerhead ribozymes in suppressing DENV infections in retrovirus transduced mosquito cells [31]. This led to the identification of several hammerhead ribozymes effective in significantly reducing DENV serotype 2 New Guinea strain (DENV2-NGC) infection of *Ae*. *albopictus* C6/36 cells. However, due to the relatively strict triplet nucleotide sequence requirements for catalysis, engineering a single hammerhead ribozyme possessing the ability to target all DENV serotypes as well as CHIKV is not practical. This necessitated exploration of ribozymes that have an increased potential for broader specificity and utility.

*Trans*-splicing group I introns provide a versatile tool for repairing defective RNA [32–44]. *Trans*-splicing group I introns have also demonstrated utility in targeting the RNA genomes of a number of viruses such as HIV-1 tat [45], cucumber mosaic virus coat protein mRNAs [32], and the hepatitis C virus internal ribosome entry site (HCV-IRES; [46,47]). In recent years, our lab has demonstrated the effectiveness of a group I intron *trans*-splicing strategy to target conserved sequences among all DENV genomes [48].

In a previous report, we noted that the 5' end of the transcript encoding our anti-DENV group I intron was composed of a 56 nt untranslated RNA leader sequence expressed from the Actin 5c promoter [49]. This observation led us to consider the possibility that additional sequences might be appended to the 5' terminus of our anti-DENV group I intron in a way that allows the recognition and catalysis of another virus genome.

This report describes the development of dual targeting group I introns for the replication suppression of all DENV serotypes and CHIKV through a “death by infection” strategy that involves the inclusion of a proapoptotic effector gene that is active only when fused to CHIKV or DENV RNA. These introns are based upon the previously described anti-DENV group I intron [48] that is modified by the addition of CHIKV targeting external and internal guide sequences, along with a corresponding interacting P10 sequence, that allows targeting of either CHIKV or DENV genomic RNAs. When coupled with an apoptosis-inducing ΔN Bax 3’ exon, these constructs *trans*-splice conserved sequences of either CHIKV or DENV to the ΔN Bax 3’ exon, leading to the induction of apoptotic cell death upon virus infection. We demonstrate antiviral and proapoptotic activity for these uniquely constructed group I introns in transformed mosquito cell cultures challenged with infectious DENV serotypes 1 through 4 and CHIKV.

The *trans*-splicing reaction of these dual targeting intron constructs was designed to attack the conserved circularization sequence (CS) of the DENV genome as well as a highly conserved region of the CHIKV NS1 gene. Splicing appends a 3’ exon RNA sequence encoding the ΔN Bax sequence to the capsid (DENV) or the NS1 (CHIKV) protein coding regions of the respective genomic RNAs resulting in translation of a chimeric protein that induces premature cell death upon infection by either DENV or CHIKV. TCID_50_-IFA analyses demonstrate suppression of each of the four DENV serotypes, and CHIKV 181/25 vaccine test strain. Results obtained from annexin V staining, effector caspase assays, and DNA ladder assays confirm that the resulting DENV CA-ΔN Bax (DCA- ΔN Bax) or CHIKV NS1- ΔN Bax (CNS1-ΔN Bax) fusion proteins induce apoptotic cell death. Our cumulative results confirm the effectiveness of our dual targeting (DENV and CHIKV) group I intron as a sequence specific antiviral that should be useful for suppression of DENV and CHIKV replication in transgenic mosquitoes.

## Results

### Basic *trans*-splicing mechanism of the group I intron

**Fig 1:**
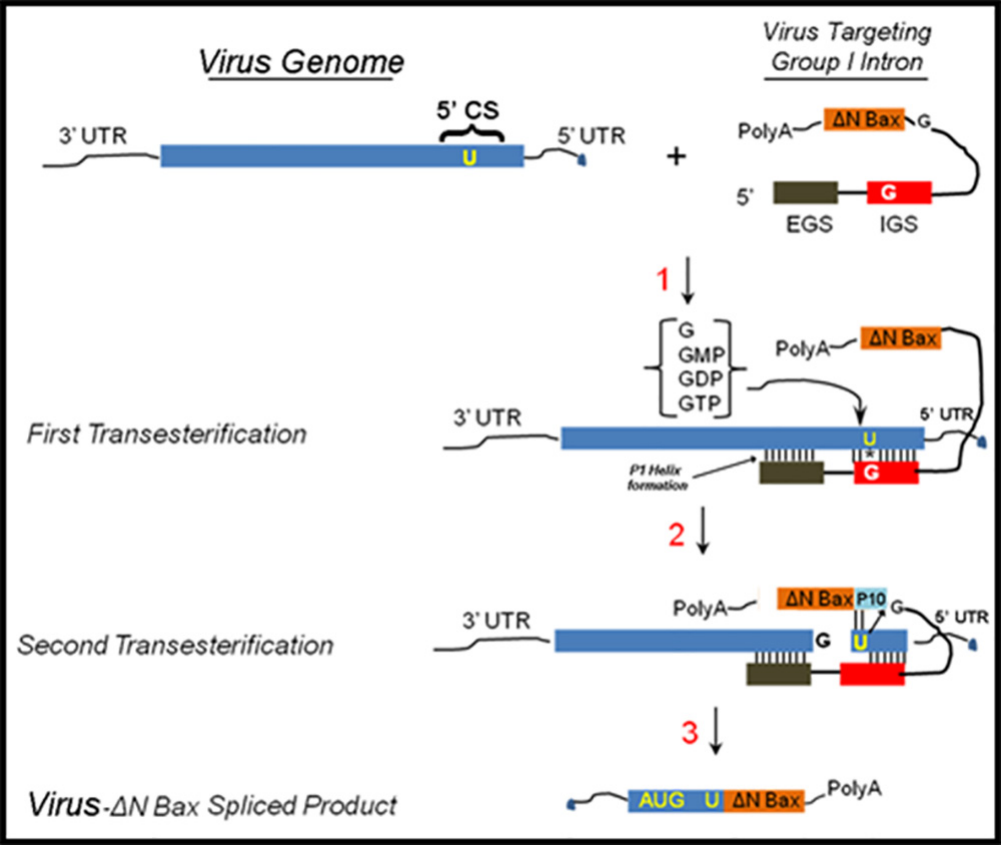
*Trans*-splicing reaction catalyzed by the group I intron. *First step →* Intron finds its target RNA sequence through complimentary base pairing with the internal and external guide sequences (IGS and EGS, respectively). The intron then sequesters the 3’-OH of a free-floating guanosine (GTP, GDP, GMP, or guanosine alone). The 3’ GNP OH attacks the phosphodiester backbone directly downstream of the reactive uracil on the 5’ exon. *Second step →* The 3’ exon is brought into proximity with the newly freed 3’-OH on the cleavage uracil, guided by the P10 helix. The 3’-OH attacks the phosphodiester backbone just upstream of the 3’ exon in another transesterification reaction, resulting in the 5’ exon and the 3’ exon being joined covalently. *Third step →* The end result is a new mRNA molecule, functional and ready for translation, formed of two separate RNA molecules. Adopted from Figure 1 in reference [49].

The *Tetrahymena thermophila* group I intron *trans*-splicing mechanism is characterized by two independent transesterification reactions ([50]; Fig 1). The ribozyme-mediated targeting of substrate RNAs forms the P1 and extended antisense helices, which promotes guanosine-mediated transesterification resulting in cleavage of the target virus RNA (Step 1). An internal guide sequence (IGS) of 9 bases complimentary base pairs with the target RNA, except a reactive guanosine that forms a wobble base pair with the targeted uracil to initiate the cleavage reaction. The external guide sequence (EGS) Watson-Crick base pairs with the substrate RNA nucleotides, downstream from the reactive uracil. This interaction provides additional stability for the *trans*-splicing reaction. Sequences from the 3’ exon (ΔN Bax) displace the distal portion of the P1 helix to form the P10 helix (Step 2). This allows the second transesterification to proceed, resulting in ligation of the DENV or CHIKV capsid and ΔN Bax. The end result is a new RNA fusion molecule that, if appropriately configured, can be translated into a new proapoptotic protein (Step 3).

Our lab has demonstrated the effectiveness of group I introns targeting sequences in the DENV 5’ CS [48]. While these group I introns efficiently cleave this conserved region, the use of these molecules as simple catalytic cleavage agents requires levels of expression that match or exceed those for the generation of virus in infected cells. Escape events may be possible in the event virus replication exceeds group I intron catalytic suppression. As a result, we presumed that expression of anti-viral group I introns alone in cells may not be the ideal method for suppressing DENV expression and decreasing the probability of generating escape mutants. We reasoned that coupling the splicing activity of the group I intron to a death-upon-infection strategy would provide an added level of insurance against the development of escape mutants. We therefore designed anti-DENV group I introns coupled with the apoptosis-inducing genes as the 3’ exon to induce cell death upon infection with DENV and validated this approach as an effective antiviral in C6/36 cells [47].

### Construction of CHIKV targeting Group I intron

**Fig 2:**
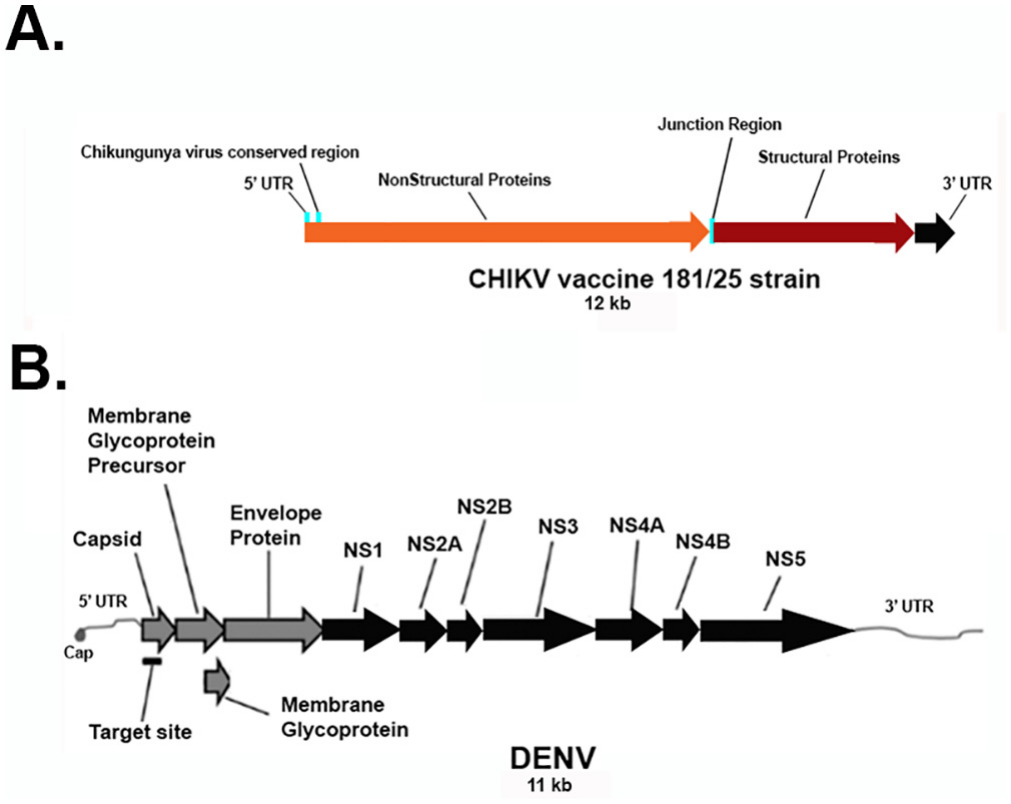
Schematic Representation of the A. CHIKV and B. DENV genomes, respectively, with regions targeted by the Dual Targeting Introns. **A**. CHIKV genomic RNA diagram with the indicated conserved region targeted for viral suppression. The CHIKV conserved domain, labeled as the CHIKV Conserved Region, was determined through a Clustal X alignment of GenBank obtained CHIKV sequences (see Materials and Methods and S5 Fig). **B**. The most invariant segments of the DENV RNA are known as the 5’ and 3’ cyclization sequences (labeled as the Group I Intron target site). These sequences are so named because they are complementary to each other and are thought to be involved in the formation of a panhandle structure during genome replication.

ClustalX alignments of available CHIKV genome sequences revealed the presence of a conserved region located within the NS1 gene of CHIKV (S5 Fig). This conserved sequence occurs in genomic forms of CHIKV RNA that are present during the early and late stages of alphavirus infection [51], and represents an ideal target for our antiviral group I strategy (Fig 2A). The anti-CHIKV group I intron was produced through PCR amplification of the anti-DENV group I intron [48,49], with a forward primer that possessed EGS and IGS sequences complimentary to nucleotides C201 to C209 and G188 to U196, respectively, of the CHIKV RNA genome. The CHIKV P10 helix forming sequences (S1 Table) and a UAA stop codon were inserted into our anti-CHIKV group I intron as previously described [48,49]. The UAA triplet was inserted immediately upstream of the UCG splice site to prevent inadvertent expression of the 3’ exon, ΔN-Bax [48,49]. Though the conserved region of the CHIKV target RNA does not possess an invariant base, a single variable base at nucleotide 152 of the DENV RNA was positioned within a non-homologous bulge loop (BL) structure that separates the IGS and EGS, and therefore did not influence the targeting of the intron (42). This BL structure promotes the formation of the P10 helix, which increases the catalytic efficiency of the intron (45).

### Construction of anti-DENV and anti-DENV/CHIKV dual targeting group I intron-ΔN Bax vectors

In previous reports, we described the construction and *trans*-splicing activity of an anti-DENV group I intron that was designed to effectively *trans*-splice all known DENV sequences by targeting the uracil at position 143 within the DENV 5’CS [48,49] (Fig 2B; see Materials and Methods). This *trans*-splicing anti-DENV group I intron included a 9 nucleotide EGS that Watson-Crick base pairs to sequences that are fully conserved among all known DENV genomes to improve catalytic efficiency and minimize potential off-target splicing interactions. A 9 base IGS was also present in this antiviral intron, possessing a reactive guanosine that forms a wobble base pair with the targeted uracil on the substrate DENV RNA (Fig 1). The anti-DENV group I intron was used in this study as a positive control for DENV *trans*-splicing and a negative control for CHIKV *trans*-splicing. It also served as a PCR template for the production of anti-CHIKV and DENV/CHIKV dual targeting group I introns.

**Fig 3:**
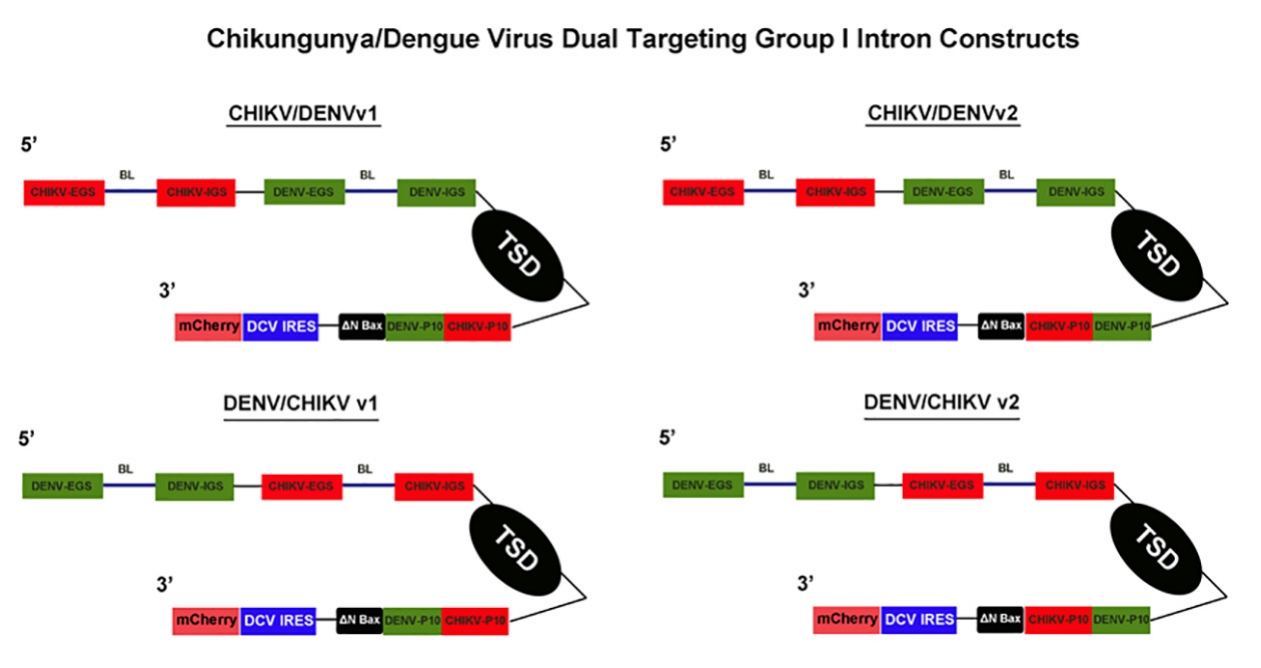
anti-CHIKV/DENV Dual Targeting Introns in a Bicistronic Configuration. Each of the *trans*-splicing dual targeting introns was tagged downstream of the ΔN Bax 3’ exon (active, further truncated version of tBax) with the mCherry fluorescent marker gene expressed from an IRES sequence of the Drosophila C Virus (DCV). Expression of these constructs was driven by the *Drosophila melanogaster* Actin 5c promoter. Each of these Dicistrovirus IRES sequences was previously determined to yield the highest levels of expression in *Ae*. *aegypti* mosquito cells [48]. The bicistronic configuration allowed monitoring for the presence and expression of these constructs within cell cultures through dual expression of antiviral intron-ΔN Bax and mCherry cistronic portions of the plasmid. Version 1 (v1) and Version 2 (v2) refer to the order of the CHIKV and DENV specific P10 helix sequences. CHIKV = Chikungunya virus targeting sequences; DENV = Dengue virus targeting sequences; IRES = internal ribosome entry site; EGS = external guide sequence; IGS = internal guide sequence; BL = bulge loop; TSD = *trans* splicing domain; P10 = P10 helix.

The antiviral group I intron constructs that we refer to as “dual targeting introns” were constructed by engineering the DENV-targeting antiviral group I intron [48,49], to include EGS, IGS, and P10 helix forming sequences specific for CHIKV (Fig 3). This was accomplished by incorporating IGS, EGS, and P10 sequences specific to the highly conserved NS1 region of the CHIKV RNA into the previously successful anti-DENV 9v1 construct [48,49], allowing targeting of genomic CHIKV RNA as well as DENV genomic RNAs by a single antivirus group I intron (Fig 3). Excluding the wobble base with the uracil at nucleotide position 143 (for DENV RNA targeting) or nucleotide position 193 (for CHIKV RNA targeting), which are required for proper cleavage [52–55], 17 bases of each set of targeting sequences from each dual targeting intron interacted directly with the intended target sequence.

The CHIKV/DENV dual targeting version 1 (v1) and version 2 (v2) group I intron constructs differ by the 5’ to 3’ arrangement of the p10 helix forming sequences that are specific for CHIKV and DENV (Fig 3). For example, the dual targeting introns with the v1 designation possess the CHIKV p10 immediately upstream (5’) of the DENV p10. The vice versa is true for the v2 dual targeting intron configurations.

The ΔN Bax coding sequence, corresponding to amino acids 112–192 of the Bax protein, was included as the 3' exon (Fig 3). The ΔN Bax protein has been shown to induce cell death in A549 and NCI-H1299 cell lines more efficiently than tBax [56]. The increased induction of apoptosis is due to deletion of the Bax BH-3 domain that facilitates protein-protein interactions between Bax and Bcl-2 or other anti-apoptotic regulators. To insure that this potent apoptosis inducer was not expressed from the intron construct, we inserted a UAA stop codon into the P9.0 helix of the group I intron immediately upstream of the UCG splice donor [48,49]. Linking the 3’exon, ΔN-Bax, to these dual targeting, antivirus group I introns was designed to insure replication suppression of two different viral RNA genomes, in this case DENV and CHIKV, with a single group I intron.

We previously constructed negative controls for *trans*-splicing activity by removing the entire catalytic core, domains P4 through P6, of the *trans*-splicing intron [48]. Using this negative control group I intron as a PCR template, we engineered two negative control group I introns, ΔCHIKV/DENVv1 and ΔDENV/CHIKVv1, as described in Materials and Methods.

The DENV/CHIKV dual targeting intron-ΔN Bax constructs were further modified by attaching a Drosophila C Virus (DCV) IRES-driven mCherry fluorescent marker gene immediately downstream of the ΔN Bax 3’ exon (Fig 3). This bi-cistronic configuration allowed monitoring for the presence and expression of the dual targeting CHIKV/DENVgroup I intron constructs within mosquito cell cultures. Expression of the entire construct was driven in mosquito cells by the *Drosophila melanogaster* Actin 5c promoter (A5c).

Dual targeting CHIKV/DENV-ΔN Bax intron constructs effectively target CHIKV and all DENV serotypes analyzed.

**Fig 4:**
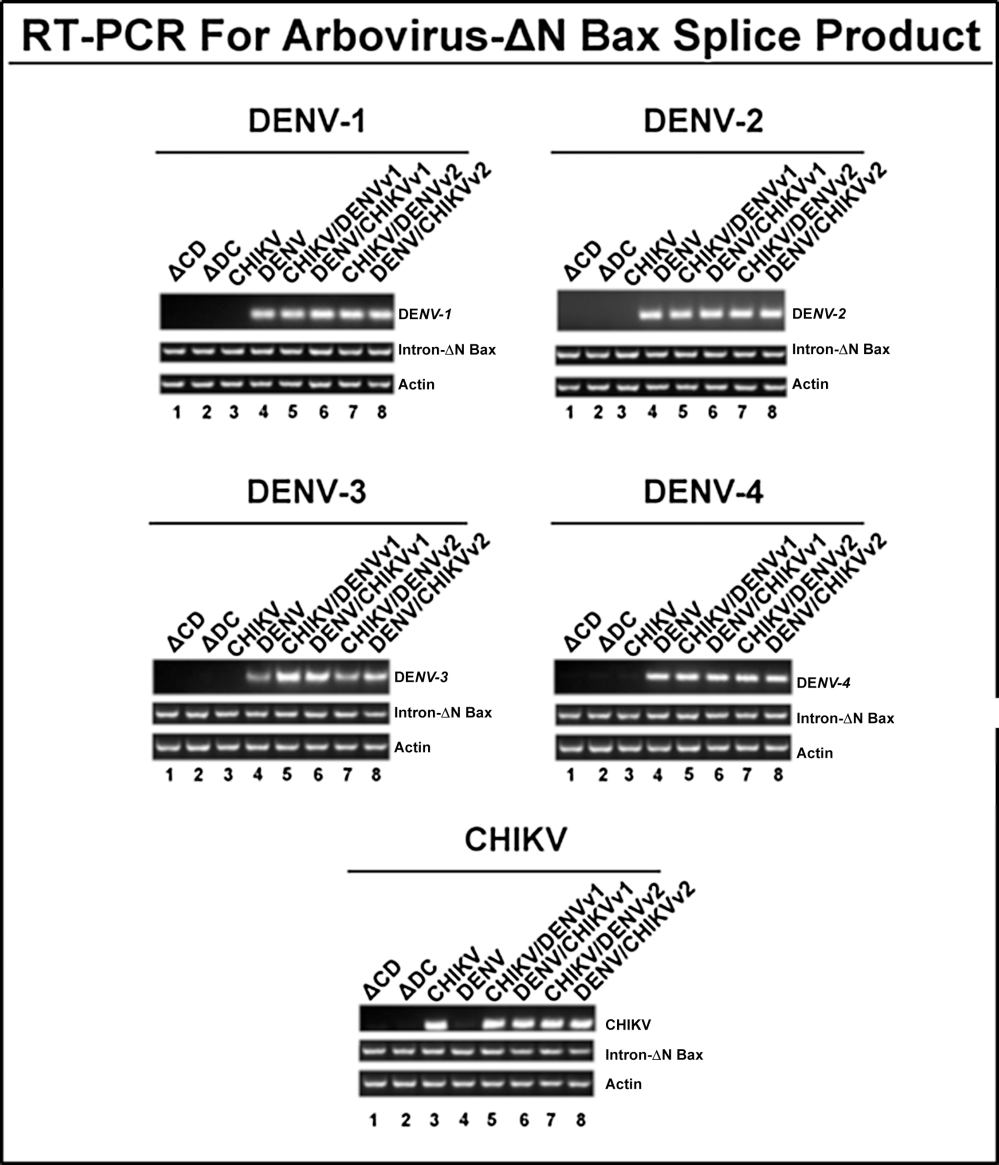
CHIKV/DENV dual targeting intron–ΔN Bax constructs effectively target CHIKV and all four DENV serotypes tested. Clonal *Ae*. *albopictus* C6/36 cells transformed with constructs bearing anti-CHIKV, anti-DENV, or the catalytically active and inactive forms of the anti-CHIKV/DENV dual targeting intron constructs were infected with one of the arboviruses indicated on each figure panel and analyzed for the presence of splice product by RT-PCR with heterologous primers as stated in Materials and Methods. The title of each figure panel indicates the virus the CHIKV/DENV dual targeting intron–ΔN Bax constructs were tested against. CHIKV/DENV-ΔN Bax or DENV/CHIKV-ΔN Bax refers to the CHIKV/DENV dual targeting *trans*-splicing group I introns that were designed for dual targeting of CHIKV and DENV. These introns are coupled to the proapoptotic ΔN Bax as the 3’ exon. ‘v1’ and ‘v2’ refer to the order of the CHIKV and DENV specific P10 helix-forming sequences, as explained in Materials and Methods ΔCHIKV/DENVv1-ΔN Bax (ΔCD) or ΔDENV/CHIKVv1-ΔN Bax (ΔCD) refer to the anti- CHIKV/DENV dual targeting introns possessing the inactive deletion mutation of the *trans*-splicing domain that is linked to the ΔN Bax 3’ exon. The deletion mutation of the *trans*-splicing domain is designed to knock out *trans*-splicing function, providing a negative control [76]. Control RT-PCR experiments were performed with primers for actin to confirm similar RNA loading. Heterolgous primers to the intron-ΔN Bax segment of the dual targeting intron construct were used to confirm the presence of our anti-CHIKV/DENV introns. All constructs are linked to the DCV IRES-mCherry configuration as shown in Fig 3. The identity of spliced products was confirmed by sequencing (Data not shown).

Our previous studies demonstrated that anti-DENV group I intron-firefly luciferase (FL) and anti-DENV group I intron-ΔN Bax constructs were capable of effectively targeting the 5’ CS region located within the RNA of all DENV serotypes [48,49]. We followed a similar protocol to examine the ability of our dual targeting constructs to effectively target, splice, and suppress both DENV and CHIKV in transformed cells (Fig 4).

Intron-expressing stable lines were generated by co-transfection of C6/36 cells with a hygromycin selectable marker plasmid along with one of the A5c promoter plasmids expressing each of the dual targeting CHIKV/DENV group I introns, or with a negative control intron coupled to the 3’ ΔN Bax exon, as described in Materials and Methods and previous reports [48,49]. Post-spliced products of approximately 300 or 310 bp were recovered as a result of targeting either DENV or CHIKV by the respective singlet antiviral introns (lanes 3 and 4), or dual antivirus intron (lanes 5 through 8) targeting of DENV or CHIKV (Fig 4). The identity of these presumptive spliced product bands was confirmed by sequencing (Data not shown).

As expected we did not detect spliced products by RT-PCR of RNAs extracted from cells transfected with the negative control dual ΔCHIKV/DENV-ΔN Bax vector constructs (ΔC/D and ΔD/C, Lanes 1 and 2, respectively; Fig 4). Moreover, DENV CA-ΔN Bax spliced product was not detected when cells transformed with the antiviral intron designed to target only CHIKV were challenged with DENV (lane 3), nor was the CHIKV NS1 ΔN Bax spliced product detected when anti-DENV-ΔN Bax transformed C6/36 cells were challenged with CHIKV (lane 4).

### CHIKV/DENV dual targeting intron-ΔN Bax constructs initiate apoptosis upon arbovirus infection

**Fig 5:**
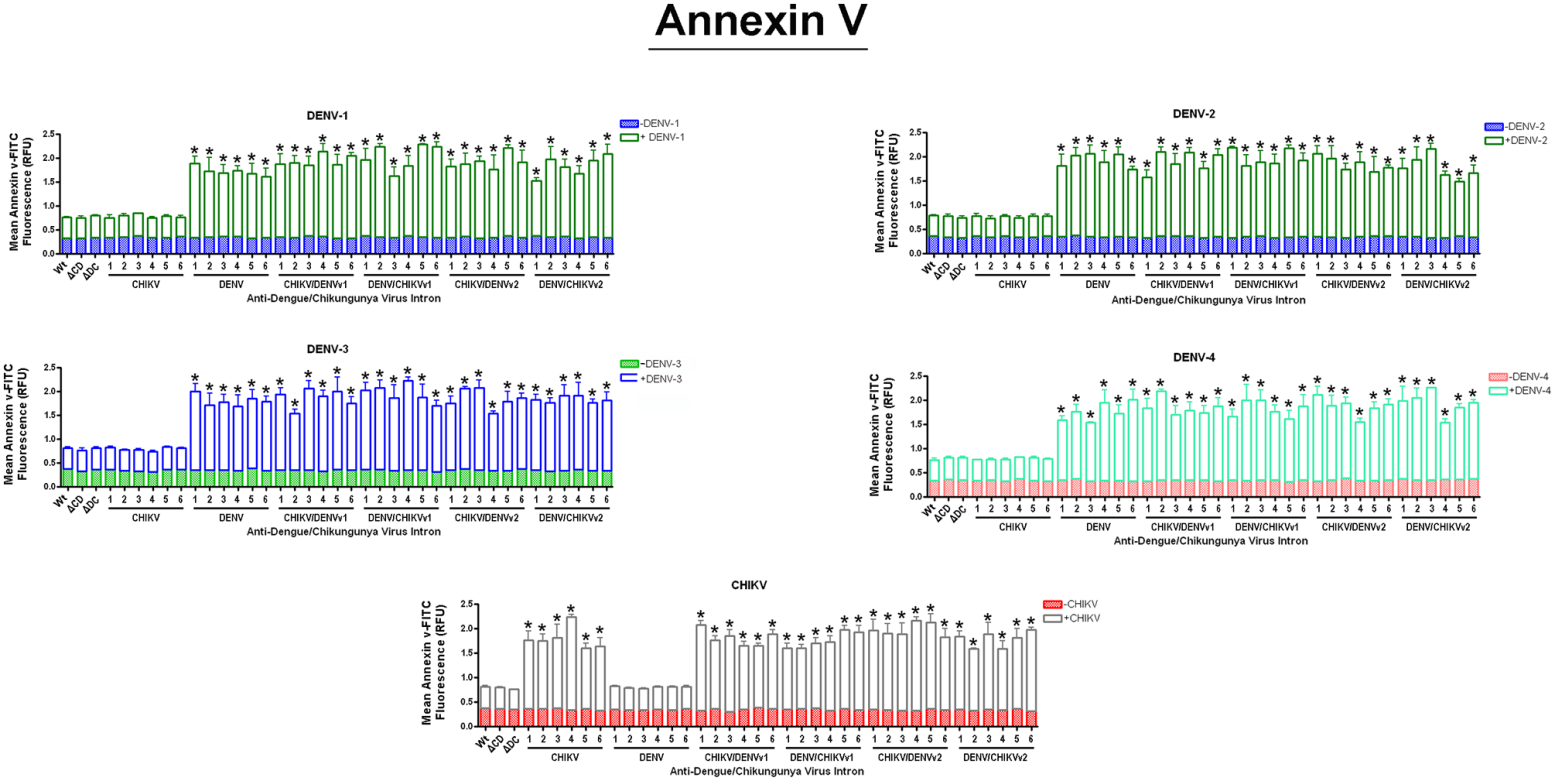
Promotion of the initial stages of apoptosis by the activation of our Dual Targeting Intron Constructs. Clonal *Ae*. *albopictus* C6/36 cells transformed with the single or dual targeting antiviral intron constructs indicated were challenged with eeach of the arboviruses shown on the figure. Cells were stained with FITC conjugated annexin V and analyzed the manufacturer’s instructions (Cayman Chemical Company; see Materials and Methods). ΔC/D- or ΔD/C refer to the anti- CHIKV/DENV dual targeting introns possessing the inactive deletion mutation of the *trans*-splicing domain that is linked to the ΔN Bax 3’ exon. Wt (wild type) refers to a negative control C6/36 cell line that does not express antiviral intron constructs. Six individual clonal cell populations per single or dual virus targeting intron construct are shown. Each number corresponds to the individual clonal cell population tested. Three wells were infected per clonal cell population and analyzed for annexin V-FITC staining. The experiment was performed at three independent times resulting in a total of nine replicates for each dual-targeting intron-ΔN Bax tested.

Annexin V apoptosis assays were performed to establish the ability of expressed DENV CA-ΔN Bax or CHIKV NS1-ΔN Bax fusion proteins to initiate apoptosis [57–59]. C6/36 clonals stably expressing CHIKV-ΔN Bax, DENV-ΔN Bax, or the dual targeting intron-ΔN Bax constructs were challenged with each DENV serotype indicated, or CHIKV strain and analyzed by annexin V staining as described in Materials and Methods (Fig 5). As a control, C6/36 cell lines stably expressing CHIKV-ΔN Bax, DENV-ΔN Bax, and CHIKV/DENV dual targeting intron-ΔN Bax constructs were tested in the absence of virus for annexin V activity to ensure that stable expression of the intron itself does not trigger apoptosis.

As expected, non-transfected (designated as "wild type") C6/36 cells, as well as clonal cells expressing the inactive anti-CHIKV/DENVv1 group I intron-ΔN Bax constructs (ΔCHIKV/DENVv1-ΔN Bax or ΔDENV/CHIKV v1-ΔN Bax), displayed background annexin V-FITC staining following DENV or CHIKV infection (Fig 5). Expression of the active dual targeting intron constructs with ΔN Bax as the 3’ exon, irrespective of the DENV serotype or CHIKV challenge, led to the activation of cellular apoptotic pathways as indicated by the binding of FITC-conjugated annexin V to the PS externalized on the cell surface [59].

### All CHIKV/DENV dual targeting-ΔN Bax clonal cell lines displayed 2 fold greater initiation of apoptosis than negative control cells

Although C6/36 cell lines stably expressing the anti-CHIKV -ΔN Bax construct did not exhibit annexin V staining above background in the presence of DENV infection, CHIKV challenge resulted in significant annexin V staining (Fig 5). Similarly, C6/36 cell lines stably expressing the anti-DENV-ΔN Bax construct did not exhibit annexin V staining above background in the presence of CHIKV infection, while DENV challenge resulted in positive staining (Fig 5). These results further demonstrate the specificity of our anti-CHIKV/DENV dual targeting intron approach.

### Activity of CHIKV/DENV dual targeting intron-ΔN Bax constructs against DENV and CHIKV results in further progression of apoptosis

Caspases are associated with the initiation of the “death cascade” and are important markers of the cell’s entry point into apoptosis [60,61]. Caspase-3 is a mammalian-specific effector caspase. However, mosquitoes possess several “effector caspases”, and it is not clear which caspase(s) is/are involved in apoptosis in *Aedes* mosquito cells [62]. Therefore, for the purposes of this paper, we will simply refer to these mosquito caspases as ‘effector caspases’.

**Fig 6:**
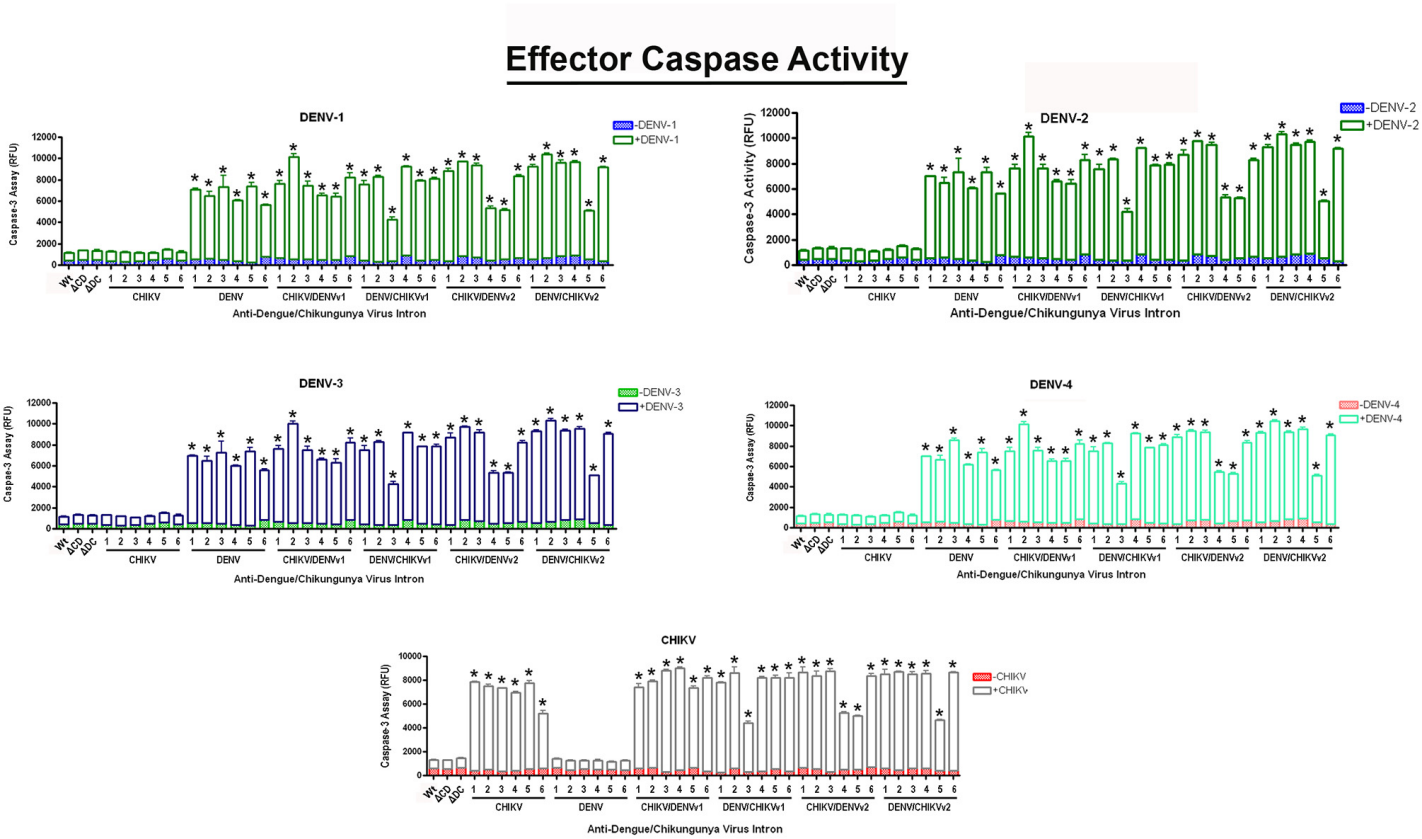
Effector caspase activation confirms the induction of apoptosis by the activation of Dual Targeting Intron Constructs. Clonal *Ae*. *albopictus* C6/36 cells transformed with the single or anti-CHIKV/DENV dual targeting antiviral intron constructs indicated were challenged with either of the four DENV serotypes or CHIKV vaccine strain 181/25 (MOI 0.01). 1x10^6^ cells were assessed for effector caspase activity, per the manufacturer’s instructions (see Materials and Methods). ΔC/D-ΔN Bax or Δ/C-ΔN Bax refer to the anti- CHIKV/DENV dual targeting introns possessing the inactive deletion mutation of the *trans*-splicing domain that is linked to the ΔN Bax 3’ exon. Wt (wild type) refers to a negative control C6/36 cell line that does not express antiviral intron constructs. Numbered lanes indicate clonal cell populations expressing single or dual virus targeting intron constructs. Three wells were infected per clonal cell population and analyzed for “effector caspase” activity. The experiment was performed at three independent times resulting in a total of nine replicates for each dual-targeting intron-ΔN Bax tested.

Clonal C6/36 cell populations expressing anti-CHIKV/DENV-ΔN Bax constructs, linked to a DCV IRES-mCherry configuration, were challenged with each of the four DENV serotypes or the CHIKV strain 181/25, and analyzed (Fig 6), as described in Materials and Methods. As a control, C6/36 cell lines stably expressing antiviral intron constructs were tested in the absence of virus for effector caspase activity to ensure that stable expression of the intron itself does not trigger apoptosis.

In the presence of either CHIKV or DENV challenge, all CHIKV/DENVv1-ΔN Bax clonal cell lines displayed 10 fold greater initiation of apoptosis than negative control cells. Five out of 6 DENV/CHIKVv1 clonal cell lines initiated apoptosis at levels similar to CHIKV/DENVv1 clonals, as was the case for 4 out of 6 for CHIKV/DENVv2 and 5 out of 6 DENV/CHIKVv2 clonal cell populations tested. As expected, wild type C6/36 or ΔCHIKV/DENVv1-ΔN Bax or ΔDENV/CHIKV v1-ΔN Bax constructs displayed only background levels of effector caspase activity, following DENV or CHIKV infection.

Uninfected wild type C6/36 displayed minimal effector caspase activity, comparable to background levels following CHIKV or DENV infection. Similar results were also observed for cell clones transformed with the active anti-DENV group I intron following CHIKV challenge, and the active anti-CHIKV group I intron following DENV challenge, demonstrating virus specificity of our anti-arbovirus approach. The absence of significant effector caspase activity observed with the inactive dual targeting introns ΔCHIKV/DENVv1-ΔN Bax or ΔDENV/CHIKV v1 following CHIKV or DENV challenge demonstrated effective suppression of read through of the ΔN Bax 3’ exon linked to these intron constructs.

**Fig 7:**
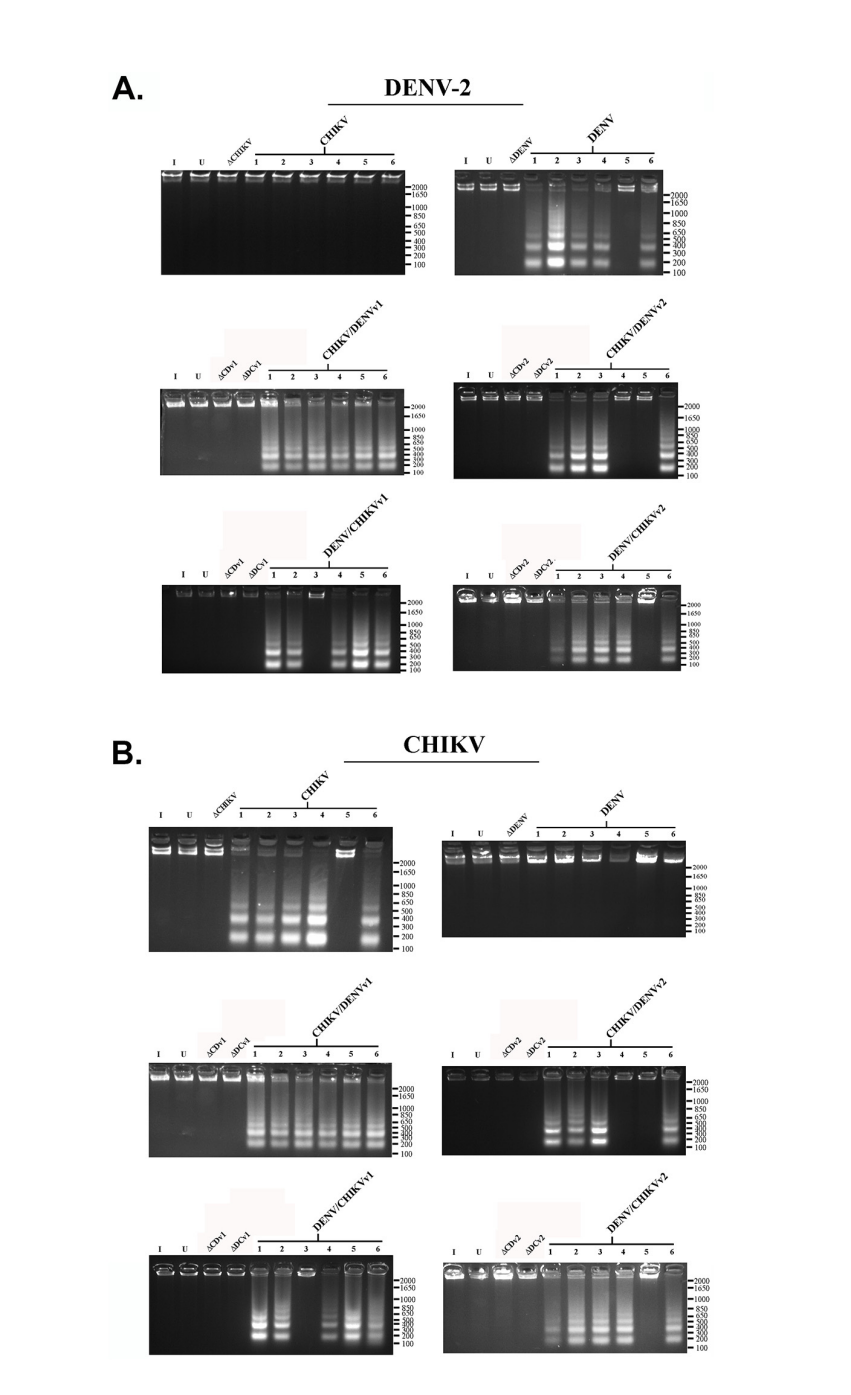
DNA Fragmentation Assay. Clonal *Aedes albopictus* C6/36 cells transformed with the anti-CHIKV, anti-DENV, anti-CHIKV/DENV or anti-DENV/CHIKV dual targeting antiviral intron constructs indicated were each challenged with each arbovirus indicated. Following virus challenge cells were pelleted, lysed, and analyzed as described in Materials and Methods. DNA fragmentation results obtained following clonal C6/36 cells challenged with **A**. CHIKV or **B**. DENV-2 are shown. See S1 Fig through S5 Fig for DNA fragmentation assays performed following challenge with DENV-1, DENV-3, DENV-4, or DNA fragmentation assays performed on uninfected clonal cells expressing anti-CHIKV, anti-DENV or anti-CHIKV/DENV dual targeting antiviral intron constructs. Numbered lanes indicate clonal cell populations expressing single or dual virus targeting intron constructs. Commercial DNA ladder served as size standards for each gel. Size of DNA ladder is indicated on the right of each gel. I = Wt C6/36 mosquito cells infected with virus indicated; U = uninfected; Wt C6/36 mosquito cells; ΔC/Dv1 = ΔCHIKV/DENVv1; ΔD/Cv1 = ΔDENV/CHIKVv1; ΔC/Dv2 = ΔCHIKV/DENVv2; ΔD/Cv2 = ΔDENV/CHIKVv2.

A hallmark of apoptosis is the degradation of nuclear DNA into nucleosomal units of approximately 180 bp in length [63], yielding a DNA ladder appearance that can be visualized on an agarose gel. DNA fragmentation analysis was performed on infected anti-CHIKV/DENV dual targeting intron-ΔN Bax expressing clonal cell lines, as described previously [64,65] and in Materials and Methods, to further demonstrate the apoptosis inducing capabilities of our antiviral constructs (Fig 7 and S1 Fig through S5 Fig).

As expected, infected or uninfected wild type C6/36 cells did not display fragmentation of nuclear DNA. Clonal *Ae*. *albopictus* C6/36 cells stably expressing the *trans*-splicing deficient ΔCHIKV/DENVv1-ΔN Bax, ΔDENV/CHIKVv1-ΔN Bax, ΔCHIKV/DENVv1-ΔN Bax, or ΔDENV/CHIKVv1-ΔN Bax constructs did not display observable DNA fragmentation upon infection with CHIKV (Fig 7A) or DENV (Fig 7B and S1 Fig through S4 Fig), further demonstrating that the insertion of a UAA codon in the P9.0 helix of the group I intron prevents premature expression of the ΔN Bax effector gene. DNA fragmentation was evident in all CHIKV/DENVv1 dual targeting intron-ΔN Bax clonal cell populations infected with CHIKV or DENV. 5 out of 6 DENV/CHIKVv1 clonal cell lines displayed DNA fragmentation similar to CHIKV/DENVv1 clonals, as was the case for 4 out of 6 for CHIKV/DENVv2 and 5 out of 6 DENV/CHIKVv2 clonal cell populations tested.

Wild type C6/36 or the inactive controls (ΔCHIKV/DENVv1-ΔN Bax, ΔDENV/CHIKV v1-ΔN Bax, ΔCHIKV/DENVv1-ΔN Bax, ΔDENV/CHIKV v1-ΔN Bax) displayed no detectable DNA fragmentation following DENV or CHIKV infection. Uninfected wild type C6/36 also displayed no DNA fragmentation. Similar results were also observed for cell clones transformed with the active anti-DENV group I intron following CHIKV challenge, and the active anti-CHIKV group I intron following DENV challenge, demonstrating the specificity of our anti arbovirus approach. The absence of observable DNA fragmentation with the dual targeting CHIKV/DENV introns, without CHIKV or DENV challenge, demonstrated effective suppression of read through of the ΔN Bax 3’ exon linked to these intron constructs.

All DNA fragmentation assay results directly correlated with the clonal cell populations displaying high levels of effector caspase activity, and helped confirm that optimal antiviral group I intron mediated DENV CA-ΔN Bax or CHIKV NS1-ΔN Bax splice formation and subsequent protein expression leads to full induction of apoptosis in the presence of arbovirus infection.

### Anti-CHIKV/DENV dual targeting intron-ΔN Bax activation leads to full suppression of DENV replication

**Fig 8:**
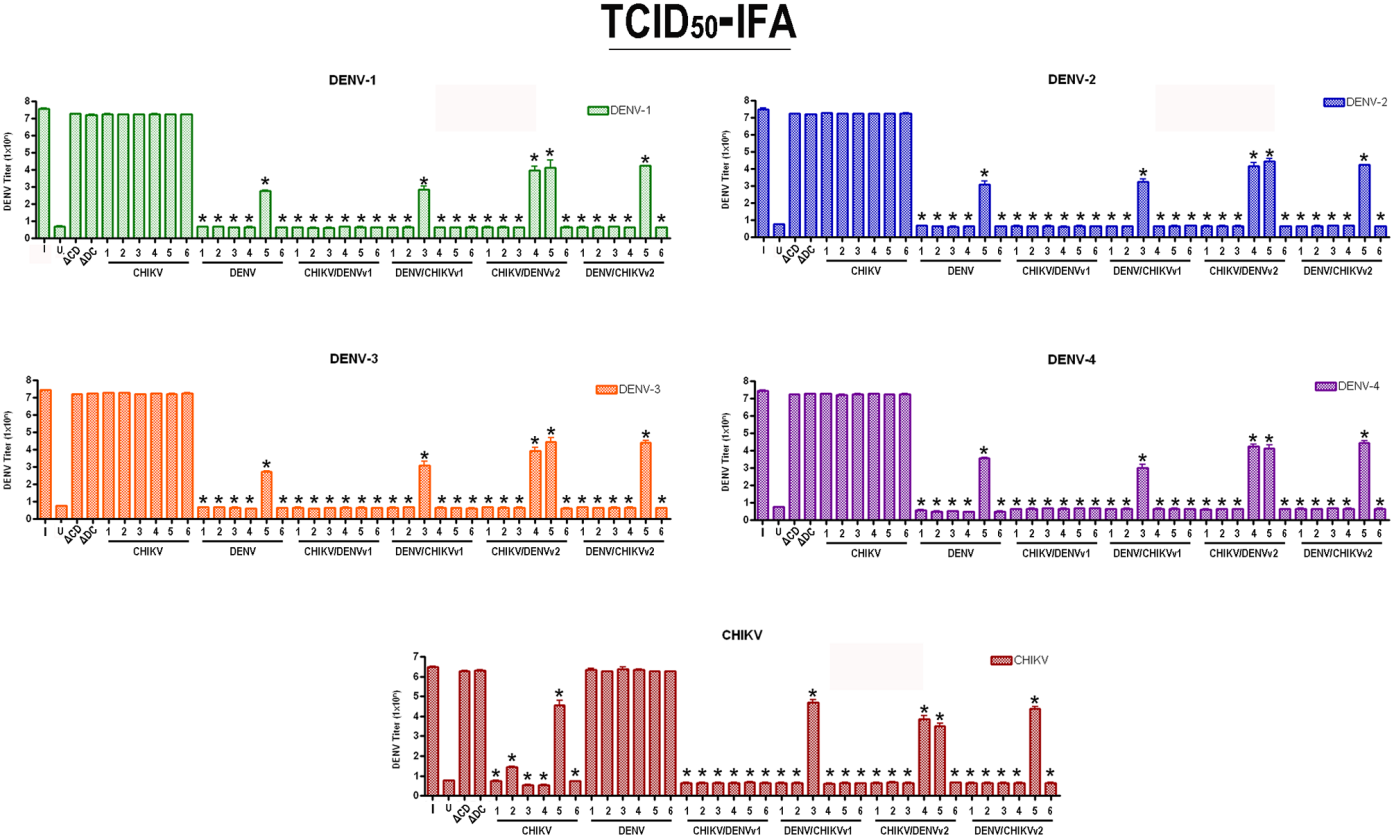
Suppression of CHIKV and DENV replication is evident in clonal cell populations expressing antiviral Dual Virus Targeting Group I Intron Constructs. Clonal *Ae*. *albopictus* C6/36 cells, transformed with the single or dual targeting antiviral intron constructs indicated, were challenged with each arbovirus indicated. Infected cell supernatants were collected, and viral titers were determined by TCID_50_-IFA. I = wild type C6/36 mosquito cells infected with virus indicated; U = uninfected wild type C6/36 mosquito cells; ΔC/D = ΔCHIKV/DENVv1; ΔD/C = ΔDENV/CHIKVv1. Supernatants originating from each clonal cell population were used as inoculum for three wells of fresh C6/36 cells and analyzed for the presence of each DENV serotype of CHIKV vaccine strain 181/25 by TCID_50_-IFA. The experiment was performed at three independent times resulting in a total of nine replicates for each dual-targeting intron-ΔN Bax tested.

Effective targeting and suppression of DENV replication was previously demonstrated through the expression of anti-DENV group I intron-FL and anti-DENV group I intron-ΔN Bax constructs [48,49]. We expected that clonal cell expression of a ΔN Bax product following DENV or CHIKV targeting by our dual-targeting intron constructs would demonstrate an enhanced suppressive effect. C6/36 cell clones stably expressing dual targeting intron constructs bearing ΔN Bax as a 3’ exon were challenged with each DENV serotype indicated or CHIKV 181/25, and virus production was quantified by TCID_50_-immunofluorescence antibody assays as described in Materials and Methods (Fig 8; [31]).

In the presence of either CHIKV or DENV challenge, all CHIKV/DENVv1-ΔN Bax clonal lines demonstrated complete suppression of DENV and CHIKV replication. More specifically, in all 6 clonal lines that stably expressed CHIKV/DENVv1, none displayed detectable levels of DENV or CHIKV, exhibiting up to 7 logs reduction in viral titer when compared to the wild type C6/36 infection control (I). 5 out of 6 DENV/CHIKVv1 clonal lines fully suppressed these arboviruses, as was the case for 4 out of 6 for CHIKV/DENVv2 and 5 out of 6 DENV/CHIKVv2 clonal cell populations tested. As expected, virus infected wild type C6/36 (I) or ΔCHIKV/DENVv1-ΔN Bax or ΔDENV/CHIKV v1-ΔN Bax constructs did not exhibit suppression of DENV or CHIKV replication. This result was also observed for clonal cells transformed with either the active anti-DENV group I intron following CHIKV challenge and active anti-CHIKV group I intron following DENV challenge.

## Discussion

Our results demonstrate, for the first time, that a single group I intron can be configured to target and *trans* splice two different RNA sequences. In this instance we establish the effectiveness of a constitutively expressed group I intron that targets and *trans*-splices conserved sequences within CHIKV and DENV, viruses representing two distantly related families of arboviruses. These *trans*-splicing, dual targeting group I intron configurations employ a 3' exon, ΔN Bax, that, when appropriately spliced with coding domains from each virus, induces apoptotic cell death upon infection in transformed mosquito cells.

Group I *trans*-splicing introns have an established potential for targeting RNA virus genomes in infected cells [45,46,48]. In previous reports we determined an optimal group I intron target sequence following an alignment of 98 instances of DENV that identified one highly conserved region positioned within the capsid coding sequence at nucleotides C131 to G151. These nucleotides are a part of the 5’-3’ CS domain of the DENV genome [66,67] that is essential for DENV replication [67] and include the amino terminal portion of the CA protein coding region. In this report we also identified a highly conserved region within the CHIKV NS1 gene at nucleotides G188 to C216 (S5 Fig) that we demonstrate serves as an effective target for *trans* splicing.

The unique configuration of our anti-CHIKV/DENV dual targeting group I introns catalyzes *trans*-splicing of the 5’ conserved target sequences of the DENV and CHIKV genomes to a 3’ ΔN Bax exon to effectively induce apoptotic death of cells following infection, thus preventing viral spread. A UAA stop codon was inserted in the *trans*-splicing domains of these introns to prevent premature expression of ΔN Bax that may result in cell death prior to infection. Following CHIKV/DENV dual targeting intron catalysis of DENV genomes at uracil 143, or CHIKV RNA genomes at U193, a chimeric mRNA is formed that consists of the 5’ cap, 5' UTR, and either 144 nucleotides of the DENV Capsid (CA) or 117 nucleotides of the CHIKV NS1 coding sequences appended to the 3’ ΔN Bax exon. These chimeric RNAs express a DENV CA-ΔN Bax or CHIKV NS1-ΔN Bax fusion protein that is demonstrated capable of inducing apoptotic cell death instead of allowing a productive virus infection.

Effective suppression of arbovirus replication with induction of apoptosis has been previously demonstrated in mosquitoes, most notably with Sindbis viruses [68]. Therefore, providing an apoptotic response in addition to genome targeting should enhance suppression of DENV or CHIKV replication as well. The ability to induce apoptosis following targeted splicing of viral genomes is an important advantage of this antiviral approach. While our group I introns demonstrated the capacity to cleave the highly conserved DENV 5’ CS region and a highly conserved region found within the CHIKV NS1 gene, the utility of these molecules as simple catalytic cleavage agents requires levels of expression that are equal to or greater than those generated by virus in infected cells.

Since escape events may arise if the rate of virus replication exceeds the rate of group I intron catalytic suppression, coupling the splicing activity of group I introns to a death-upon-infection strategy provides an added level of insurance against the development of escape mutants by insuring virus replication rates do not exceed group I intron expression and catalytic rates.

The potential emergence of escape mutants resulting from changes in the viral RNA, preventing targeting by antiviral ribozymes, is a possibility. The potential inability of the CHIKV/DENV dual targeting group I introns to suppress either CHIKV or DENV escape mutants can occur if the ribonucleic acid residues that constitute the IGS are not able to successfully target CHIKV or DENV RNA. This can be due to a mutation of the targeted uracil or the viral RNA residues targeted by the IGS. Mutation of viral RNA residues targeted by the EGS may not have a detrimental effect on group I intron catalysis of the viral RNA genome. It has been demonstrated that mutation of any residue in the sequence targeted by the EGS domain of group I introns does not eliminate catalysis or *trans*-splicing of the viral RNA sequences to the 3’ exon RNA.

We constructed a set of anti-arbovirus group I *trans*-splicing intron constructs (S1 Table, Fig 3) that are coupled to the proapoptotic ΔN Bax 3’ exon to assess whether the relative order of virus specific targeting sequences and P10 helices in these constructs affected activity against each virus. The results confirm that these constructs effectively *trans*-splice the RNA of an infecting DENV or CHIKV to the ΔN Bax 3’ exon when constitutively expressed as RNA in transformed C6/36 cells, irrespective of the relative position of the targeting sequences. These intron constructs effectively suppress DENV or CHIKV infection of transformed mosquito cells. Clonal C6/36 cell populations expressing the anti-CHIKV/DENV group I intron-ΔN Bax constructs, linked with the IRES driven mCherry as a bicistronic transcript (Fig 3), resisted infection with either CHIKV or DENV. Furthermore, addition of these IRES/mCherry configurations immediately downstream of the 3’ ΔN Bax exon did not appear to alter the *trans*-splicing capabilities of these *trans*-splicing introns (Fig 4), or affect the ability of ΔN Bax to initiate apoptosis in DENV or CHIKV infected cells (Figs 5, 6 and 7).

The expression and pro-apoptotic function of ΔN Bax is not inhibited by the 15 amino acids of CHIKV NS1 or 19 amino acids of dengue CA proteins fused to its N-terminus. Expression and activity of ΔN Bax, CNS1- ΔN Bax or DCA-ΔN Bax expressed in cells are not significantly different, and *trans*-splicing of the CHIKV and DENV RNA genomes by CHIKV-ΔN Bax (against CHIKV RNA), DENV-ΔN Bax (against DENV RNA), or CHIKV/DENV-ΔN Bax leads to the activation of cellular apoptosis as indicated by annexin V-FITC (Fig 5), effector caspase assays (Fig 6), and DNA ladder analysis (Fig 7 and S1 Fig through S5 Fig). None of these assays indicate apoptotic cell death when the *trans*-splicing negative ΔCHIKV/DENV or ΔDENV/CHIKV intron-bearing constructs are expressed or when cultured wild type C6/36 mosquito cells are infected with either CHIKV or DENV, confirming our results are a consequence of the presence of a *trans*-spliced RNA encoding the CNS1-ΔN Bax or DCA-ΔN Bax. Results obtained also demonstrate that fusion of the N-terminal amino acids of the DENV CA or CHIKV NS1 proteins to the N-terminus of the ΔN Bax protein does not eliminate the apoptotic inducing capacity of ΔN Bax, as may have been expected. The C-terminal residues of Bax possess the pore forming function of this pro-apoptotic protein [69], and may be the reason ΔN Bax retains proapoptotic activity in the presence of an N-terminal addition. Other researchers have analyzed N-terminal epitope-tagged variants of tBax with little alteration in activity [56,70,71].

Lower effector caspase and inhibition of viral replication may be due to the hindrance of dual targeting intron-ΔN Bax mRNA translocation out of the nucleus, leading to a decrease in the number of trans-spliced mRNA molecules that are produced following dual targeting intron-ΔN Bax targeting of CHIKV or DENV RNA in the cytoplasm of clonal cells.

Several labs have demonstrated that differences observed between annexin V and effector caspase/DNA fragmentation assays, may reflect the fact that the early stages of apoptosis can be reversed [72–76], preventing the progression of several clonal cell populations from the early stages of apoptosis (positive annexin V staining) to the latter stages of apoptosis (negative effector caspase activity and DNA fragmentation). Additionally, variability seen among the individual clones can be attributed to differences in expression of the group I introns due to position effects resulting from random integration of the transgene.

Additional support for the utility of these anti-CHIKV/DENV dual arbovirus targeting constructs as potent antiviral effectors is evidenced by the TCID_50_-IFA results that demonstrate suppression of infectious virus production from transformed clonal cell lines upon challenge with either CHIKV or each of the four serotypes tested (Fig 8). These results validate the utility of this single antiviral effector gene as a potential means for producing transgenic mosquitoes that will suppress DENV and CHIKV transmission.

We now have a set of anti-CHIKV/DENV group I introns that each have the ability to target four DENV serotypes and CHIKV. Our results demonstrate for the first time that group I introns may be configured to mediate *trans*-splicing against two different RNA targets. In this case, we successfully used this approach to create antiviral group I intron constructs that could arm mosquito cells to suppress two mosquito-borne viruses (CHIKV and DENV). Targeting these co-endemic viruses with a single catalytic ribozyme eliminates the necessity to construct and test separate catalytic RNAs or siRNA molecules, and could greatly facilitate the establishment of effective transgenic mosquito lines for potential release. The induction of cellular apoptosis by our anti-CHIKV/DENV constructs following DENV or CHIKV *trans*-splicing reduces the opportunity for escape mutants that may evolve in the infected cell and prevents the virus replication from overriding catalytic intron activity if the replication of DENV or CHIKV supersedes the catalytic activity of the anti-CHIKV/DENV group I intron.

## Materials and Methods

### Cells, Virus and Antibody

The *Ae*. *albopictus* C6/36 cells used in this study were obtained from ATCC, and maintained in Leibovitz’s L-15 media (Atlanta Biologicals) supplemented with 10% FBS (Atlanta Biologicals), 10% TPB (triptose phosphate broth; Invitrogen/Gibco), penicillin G (100U/ml; Invitrogen/Gibco) and streptomycin (100μU/ml; Invitrogen/Gibco), and grown in a 28°C incubator and passaged every 4 days. Assays involving DENV and CHIKV infections required L-15 media supplemented with 2% FBS and 10% TPB. Viral stocks were prepared as previously described [31,48,77].

CHIKV and DENV sequence data were provided by NCBI. Genbank GenInfo identifiers for the four DENV serotypes and CHIKV used in this study comprise the following: DENV type 1 Hawaii: DQ672564.1, DENV type 2 strain New Guinea C (NGC): AF038403.1, DENV type 3 strain ThD3 0010 87(strain H87): AY676352.1, DENV 4 strain DENV-4/SG/06K2270DK1/2005 (strain H241): GQ398256.1, and CHIKV vaccine strain 181/25: L37661.

TCID_50_-IFA analyses involving DENV infection were performed using monoclonal antibody (MAb) 4G2 (kindly provided by Dr. Stephen Higgs, Kansas State University), while analyses involving CHIKV infection were performed using a Chikungunya 181/25 vaccine strain specific antibody (IBT Bioservices, Gaithersburg, Maryland, USA).

### Plasmid construction

Assurance of identity and integrity of all plasmids used was established through sequencing and restriction analysis. All restriction enzymes were obtained from New England Bio Labs (NEB).

The *Drosophila melanogaster* actin 5c (A5c) promoted anti- DENV 9v1 *trans*-splicing intron construct employed in this study was used previously to *trans*-splice DENV type 2-NGC targets with either the firefly luciferase (FL) [48] or ΔN Bax [49] 3’exons, and was used as a template for the production of anti-CHIKV/DENV dual targeting *trans*-splicing introns by PCR amplification. Our negative controls for *trans*-splicing activity, ΔCHIKV/DENVv1 and ΔDENV/CHIKVv1, were produced by a two-step PCR amplification process. First, the entire catalytic core [52], domains P4 through P6 [78], of the anti-DENV group I intron were removed by PCR amplification with Platinum Taq polymerase (Invitrogen) using the forward and reverse primers listed in S2 Table. The PCR product was used to replace the catalytic core of the anti-DENV group I intron using the enzymes *MluI* and *NheI*. Then, the resulting inactive intron was used as a template to produce ΔCHIKV/DENVv1 and ΔDENV/CHIKVv1 negative control introns using forward and reverse primers that possess the viral targeting sequences and P10 helix forming sequences (S2 Table). This resulted in control introns that lacked *trans*-splicing activity.

Group I introns that possess efficacy in targeting of CHIKV and DENV were produced by PCR amplification of the anti-DENV group I intron previously described [48]. See S2 Table for sequences of primers used to produce these dual arbovirus targeting introns. Amplification of the CHIKV/DENV introns was achieved with forward primers possessing the IGS and EGS complementary to the RNAs of CHIKV and DENV, respectively, were tailed with the *MluI* restriction site sequence. The dual arbovirus targeting introns referred to as DENV/CHIKV were produced by PCR amplification with forward primers possessing IGS and EGS complementary to the RNAs of DENV and CHIKV, respectively, were tailed with the *MluI* restriction site sequence.

Any active dual targeting CHIKV/DENV dual targeting intron listed with a “version 1” (i.e. v1) or “version 2” (i.e. v2) designation were each produced by a 2 step PCR process. Version 1 (v1) introns possess 6 nucleotides responsible for the formation of the CHIKV specific P10 helix immediately followed by 6 nucleotides responsible for the formation of the DENV specific P10 helix (Fig 3). Production of these “v1” introns was achieved by PCR with each respective forward primer possessing either CHIKV/DENV or DENV/CHIKV targeting sequences and a reverse primer possessing the CHIKV P10 helix forming sequences (also functions as a *PstI* site), and tailed with a *XhoI* site that is immediately upstream of the *PstI* restriction site/CHIKV P10 helix (S2 Table). The *XhoI* site was removed, and DENV P10 helix forming sequences inserted, by PCR amplification of the ΔN Bax 3’ exon with forward and reverse primers tailed with *PstI* and *XbaI*, respectively. This final PCR step resulted in insertion of DENV P10 helix forming sequences immediately upstream of the ΔN Bax 3’ exon and immediately downstream of the CHIKV P10 helix forming sequences.

Version 2 (v2) introns possess 6 nucleotides responsible for the formation of the DENV specific P10 helix immediately followed by 6 nucleotides responsible for the formation of the CHIKV specific P10 helix (Fig 3). Production of these v2 introns was achieved by PCR with each respective forward primer possessing either CHIKV/DENV or DENV/CHIKV targeting sequences and a reverse primer possessing the DENV P10 helix forming sequences immediately followed by CHIKV P10 helix forming sequences (also a *PstI* site), and tailed with a *XhoI* site that is immediately upstream of the *PstI* restriction site/CHIKV P10 helix (S2 Table). The *XhoI* site was removed by PCR amplification of the ΔN Bax 3’ exon with forward and reverse primers tailed with *PstI* and *XbaI*, respectively.

The anti-CHIKV group I intron was produced through PCR amplification of the anti-DENV group I intron [48] with a forward primer that possessed the EGS and IGS sequences corresponding nucleotides that are complimentary to nucleotides C201 to C209 and G188 to U196, respectively, of the CHIKV RNA genome and tailed with *MluI*. The CHIKV P10 helix forming sequences were inserted immediately downstream of the P9.0 helix. Production of these anti-CHIKV introns was achieved by PCR with a forward primer possessing CHIKV targeting sequences and a reverse primer possessing the CHIKV P10 helix forming sequences (also a *PstI* site), and tailed with a *XhoI* site that is immediately upstream of the *PstI* restriction site/CHIKV P10 helix (S2 Table). The *XhoI* site was removed by PCR amplification of the ΔN Bax 3’ exon with forward and reverse primers tailed with *PstI* and *XbaI*, respectively.

The anti-DENV intron construct used in this report as a negative control for CHIKV and a positive control for DENV targeting and *trans*-splicing is the same antiviral intron as the previously tested 9v1 intron [48], but was renamed “U143” for clarity [49].

Production of anti-CHIKV, anti- DENV, and anti-CHIKV/DENV dual targeting group I intron constructs possessing the DCV intergenic IRES site driving an mCherry fluorescent marker was achieved by subcloning DCV-mCherry from the previously described 9v1 construct into the antiviral group I intron constructs described above using *NotI* and *SacI* restriction sites ([48,49]; Fig 3).

### Establishment of clonal cell populations

Clonal cell populations were produced as previously described [79]. Briefly, C6/36 cells stably expressing anti-CHIKV, anti-DENV, CHIKV/DENV dual targeting intron constructs, or the inactive intron versions ΔCHIKV/DENVv1 or ΔDENV/CHIKVv1 were grown to 1x10^4^ cells/mL and then diluted to 5 cells/mL. 100μl of this cell suspension was placed in each of a 96 well plate and grown to confluency. Twelve wells of each plate were scraped and transferred to individual wells of a 24 well plate. Once confluent, cells were then transferred to a 12 well plate, then a 6 well plate, and lastly T-25 flasks. Following each transfer step, cells were maintained with 1mL L-15 complete media supplemented with 100 μg/mL hygromycin. In order to guarantee clonability, 3 cloning cycles were carried out.

### Reverse Transcription-PCR of DENV-ΔN Bax Splice Products Derived from Cell Culture

Intron-expressing stable lines were generated by co-transfection of C6/36 cells with a hygromycin selectable marker plasmid and one of either A5c promoter plasmids expressing dual targeting CHIKV/DENV group I introns or negative control introns coupled to the 3’ ΔN Bax exon, as previously described [48,49]. Following hygromycin selection, the transformed C6/36 cells were seeded at a density of 5x10^6^ cells/ml in T25 flasks and were challenged with either DENV (MOI = 0.1) or CHIKV (MOI = 0.01). Total cellular RNAs were isolated at 96 hours post-infection and assessed for CHIKV/DENV group I intron activity by RT-PCR detection of *trans*-spliced products. Post-spliced products of approximately 300 or 310 bp were recovered as a result of DENV or CHIKV targeting by the respective singlet antiviral intron (lanes 3 and 4) and dual antivirus intron (lanes 5 through 8) targeting of DENV or CHIKV (Fig 4). The identity of these presumptive spliced product bands was confirmed by sequencing (Data not shown).

Extraction of RNA from CHIKV or DENV infected and uninfected cells was performed using the Qiashredder and RNeasy Mini kits (QIAGEN Inc., Valencia, CA, USA). Extracted RNAs (5 μg) were treated with Turbo DNA-free DNAse (Applied Biosystems/Ambion, Inc. Austin, TX USA). RT-PCR was performed using the SuperScript III One-Step RT-PCR kit (Invitrogen) as directed. cDNA synthesis and PCR amplification were also performed as previously indicated [48,49].

A forward primer with the sequence 5’-GTGGACATAGACGCTGACA-3’ and a reverse primer to ΔN Bax with the sequence 5’-CACTCCCGCCACAAAGATG-3’ were used for the RT-PCR of CHIKV NS1-ΔN Bax (CNS1-ΔN Bax) splice products. For the RT-PCR of DENV CA-ΔN Bax (DCA-ΔN Bax) splice products, previously described [49] forward primers specific to each DENV serotype were used with a reverse primer possessing the sequence 5’-CACTCCCGCCACAAAGATG-3’.

### Annexin V Assay

Binding of annexin V to translocated phospholipid phosphotidylserine (PS) allows for the detection and analysis of apoptotic cells [57–59]. These assays were performed using the *Enhanced Apoptosis Kit* as indicated by the manufacturer (Cayman Chemical Co.) with a few modifications. Briefly, C6/36 clonal cell lines stably expressing the CHIKV-, DENV-, CHIKV/DENV, and DENV/CHIKV dual targeting intron-ΔN Bax bearing constructs were infected with DENV serotypes 1 through 4 (MOI = 0.1) or CHIKV strain 181/25 (MOI = 0.01) and assayed for annexin V binding at 24 hpi (with CHIKV) or 48 hpi (with DENV). 1x10^6^ clonal cells were scraped and placed in a well of a 96 well black opaque microtiter plate in triplicate for each clonal cell type assayed. FITC-annexin V stained microtiter plates were assayed for FITC-annexin V binding at 485 nm with the Spectra max M2 luminometer (Molecular Devices) and analyzed with Softmax Pro 5.4.5.

Three wells per clonal cell population indicated were infected with the arbovirus shown and analyzed for annexin V-FITC staining. This experiment was performed three times to give a total of nine replicates for each dual -targeting intron-ΔN Bax tested. Assay were performed in triplicate. Error bars indicate standard deviation of three independent experiments.

### Effector Caspase Assay

Further validation of apoptosis induction was performed by assaying for increases in effector caspase and other DEVD-specific protease activities using the *EnzChekCaspase-3 Assay Kit #2 Kit* (Life Technologies) as directed by the manufacturer. Briefly, C6/36 clonal cell lines stably expressing the CHIKV-, DENV-, CHIKV/DENV, and DENV/CHIKV dual targeting intron-ΔN Bax bearing constructs were infected with DENV serotypes 1–4 (MOI = 0.1) or CHIKV strain 181/25 (MOI = 0.01) and assayed for effector caspase activity at 4d.pi (for DENV) or 3dpi (for CHIKV). 1x10^6^ clonal cells were lysed, cell debris was pelleted, and lysates were placed in a well of a 96 well black opaque microtiter plate in triplicate for each clonal cell type assayed. Following addition of the Z-DEVD–R110 substrate, microtiter plates were assayed for effector caspase activity at 496 nm with the Spectra max M2 luminometer (Molecular Devices) and analyzed with Softmax Pro 5.4.5.

Three wells per clonal cell population indicated were infected with the arbovirus shown and analysed for “effector caspase” activity. This experiment was performed three times to give a total of nine replicates for each dual-targeting intron-ΔN Bax tested.

### DNA Fragmentation Assay

Clonal C6/36 cells (1x10^7^) stably expressing the CHIKV-, DENV-, CHIKV/DENV or DENV/CHIKV dual targeting intron-ΔN Bax constructs were infected with one of four known DENV serotypes (MOI = 0.1) or CHIKV strain 181/25 (MOI = 0.01) and assayed for DNA fragmentation as previously described [49]. Briefly, at 4 dpi (for DENV) or 3dpi (for CHIKV) infected and uninfected cells were scraped, pelleted and lysed overnight at 50°C in lysis buffer [1.67mg/ml Proteinase K, 10mM Tris (pH8.0), 100mM NaCl, 0.5% SDS 25mM EDTA]. Genomic DNA was extracted with 200 μl Phenol:Chloroform:IAA (25:24:1) and sodium acetate/ethanol precipitated. DNA pellets were resuspended in 20 μl TE buffer, RNase A treated (6.0 mg/ml) at 37°C for 3 hours, and analyzed by 2% agarose gel electrophoresis at 5v/cm and visualized under UV light. DNA fragmentation is demonstrated by the appearance of a DNA ladder-like pattern.

### TCID_50_-IFA analysis of dengue viruses

Immunofluorescence detection of cell surface expressed CHIKV or DENV envelope (E) proteins in C6/36 cultures infected with serial 10 fold dilutions were used to assess the titer of CHIKV (MOI 0.01) or each DENV serotype (MOI 0.1) tested as previously described [31,48]. 10 fold serial dilutions of infected C6/36 cell culture supernatants were harvested at 24 hpi (CHIKV) or 48 hpi (DENV) and used as inoculum for 96 well plate cultures of C6/36 cells. Plates were incubated for 4 days at 28°C without CO_2_, washed, fixed with acetone:DPBS (3:1), and stained with a primary DENV E antibody (1:200) [80], followed by a biotinylated-streptavidin detection system conjugated with FITC (Amersham Biosciences, Piscataway, NJ). Wells displaying cellular fluorescence were scored as positive for DENV infection. The number of positive wells were counted and the virus titers calculated according to Karber’s method [81].

Supernatants originating from each clonal cell population indicated were used as inoculum for three wells of naïve C6/36 cells and analyzed for the presence of each DENV serotype or CHIKV vaccine strain 181/25 by TCID_50_-IFA. This experiment was performed three times to give a total of nine replicates for each dual-targeting intron-ΔN Bax tested.

## Supporting Information

S1 Fig Alignment of CHIKV Genomic Sequences for Identification of a Fully Conserved Target Site. An alignment was performed of twenty five (25) CHIKV genomic sequences to determine the most optimal regions for the design of the chikungunya virus specific and DENV/CHIKV dual targeting antiviral group I introns by determining the region with the greatest conservation within the CHIKV RNA genomes. Nucleotide sequences in yellow indicate complete conservation. Nucleotide sequences in blue indicate partial conservation. Nucleotide sequence position is indicated at the top of the figure. GenBank Accession Numbers at the left of the figure indicate the CHIKV sequences aligned.
(TIF)

S2 Fig DNA Fragmentation Assay in the presence of DENV-1 challenge. Clonal *Ae*. *albopictus* C6/36 cells transformed with the anti-CHIKV, anti-DENV or anti-CHIKV/DENV dual targeting antiviral intron constructs indicated were each challenged with dengue virus serotype 1, processed, and analyzed as described for Fig 7 and in Materials and Methods. I = Wt C6/36 mosquito cells infected with virus indicated; U = uninfected Wt C6/36 mosquito cells; ΔC/Dv1 = ΔCHIKV/DENVv1; ΔD/Cv1 = ΔDENV/CHIKVv1; ΔC/Dv2 = ΔCHIKV/DENVv2; ΔD/Cv2 = ΔDENV/CHIKVv2.
(TIF)

S3 Fig DNA Fragmentation Assay in the presence of DENV-3 challenge. Clonal *Ae*. *albopictus* C6/36 cells transformed with the anti-CHIKV, anti-DENV or anti-CHIKV/DENV dual targeting antiviral intron constructs indicated were each challenged with dengue virus serotype 3, processed, and analyzed as described for Fig 7 and in Materials and Methods. I = Wt C6/36 mosquito cells infected with virus indicated; U = uninfected Wt C6/36 mosquito cells; ΔC/Dv1 = ΔCHIKV/DENVv1; ΔD/Cv1 = ΔDENV/CHIKVv1; ΔC/Dv2 = ΔCHIKV/DENVv2; ΔD/Cv2 = ΔDENV/CHIKVv2.
(TIF)

S4 Fig DNA Fragmentation Assay in the presence of DENV-4 challenge. Clonal *Ae*. *albopictus* C6/36 cells transformed with the anti-CHIKV, anti-DENV or anti-CHIKV/DENV dual targeting antiviral intron constructs indicated were each challenged with dengue virus serotype 4, processed, and analyzed as described for Fig 7 and in Materials and Methods. I = Wt C6/36 mosquito cells infected with virus indicated; U = uninfected Wt C6/36 mosquito cells; ΔC/Dv1 = ΔCHIKV/DENVv1; ΔD/Cv1 = ΔDENV/CHIKVv1; ΔC/Dv2 = ΔCHIKV/DENVv2; ΔD/Cv2 = ΔDENV/CHIKVv2.
(TIF)

S5 Fig DNA Fragmentation Assay in the absence of virus challenge. Clonal *Ae*. *albopictus* C6/36 cells transformed with the anti-CHIKV, anti-DENV or anti-CHIKV/DENV dual targeting antiviral intron constructs indicated were each mock infected, processed, and analyzed as described for Fig 7 and in Materials and Methods. I = Wt C6/36 mosquito cells infected with virus indicated; U = uninfected Wt C6/36 mosquito cells; ΔC/Dv1 = ΔCHIKV/DENVv1; ΔD/Cv1 = ΔDENV/CHIKVv1; ΔC/Dv2 = ΔCHIKV/DENVv2; ΔD/Cv2 = ΔDENV/CHIKVv2.
(TIF)

S1 Table Sequence composition of antiviral group I intron domains. The ribonucleotide sequences of each antiviral group I intron are shown. The left column lists the viruses targeted by the dual targeting introns containing respective targeting sequences shown. Targeting sequences specific for each virus are indicated. See methods for description of assembly. CHIKV = Chikungunya virus targeting sequences; DENV = Dengue virus targeting sequences; EGS = external guide sequence; IGS = internal guide sequence; BL = bulge loop; TSD = *trans* splicing domain; P10 = P10 helix.
(TIF)

S2 Table Primers used for Dual Targeting Intron Construct Construction. Listed are the forward and reverse primer sets used to produce the PCR fragments of anti-CHIKV/DENV introns, negative controls, and the ΔN Bax 3’ exon for plasmid insertion. Restriction sites used are indicated by lowercase nucleic acids. See Materials and Methods for description of vector construct assembly.
(TIF)
