# Supplementary figures and images for: Suppression of the Arboviruses Dengue and Chikungunya Using a Dual-Acting Group-I Intron Coupled with Conditional Expression of the Bax C-Terminal Domain
Source: PLoS One. 2015 Nov 18;10(11):e0139899. doi: 10.1371/journal.pone.0139899 (PMC4651551; doi:10.1371/journal.pone.0139899)

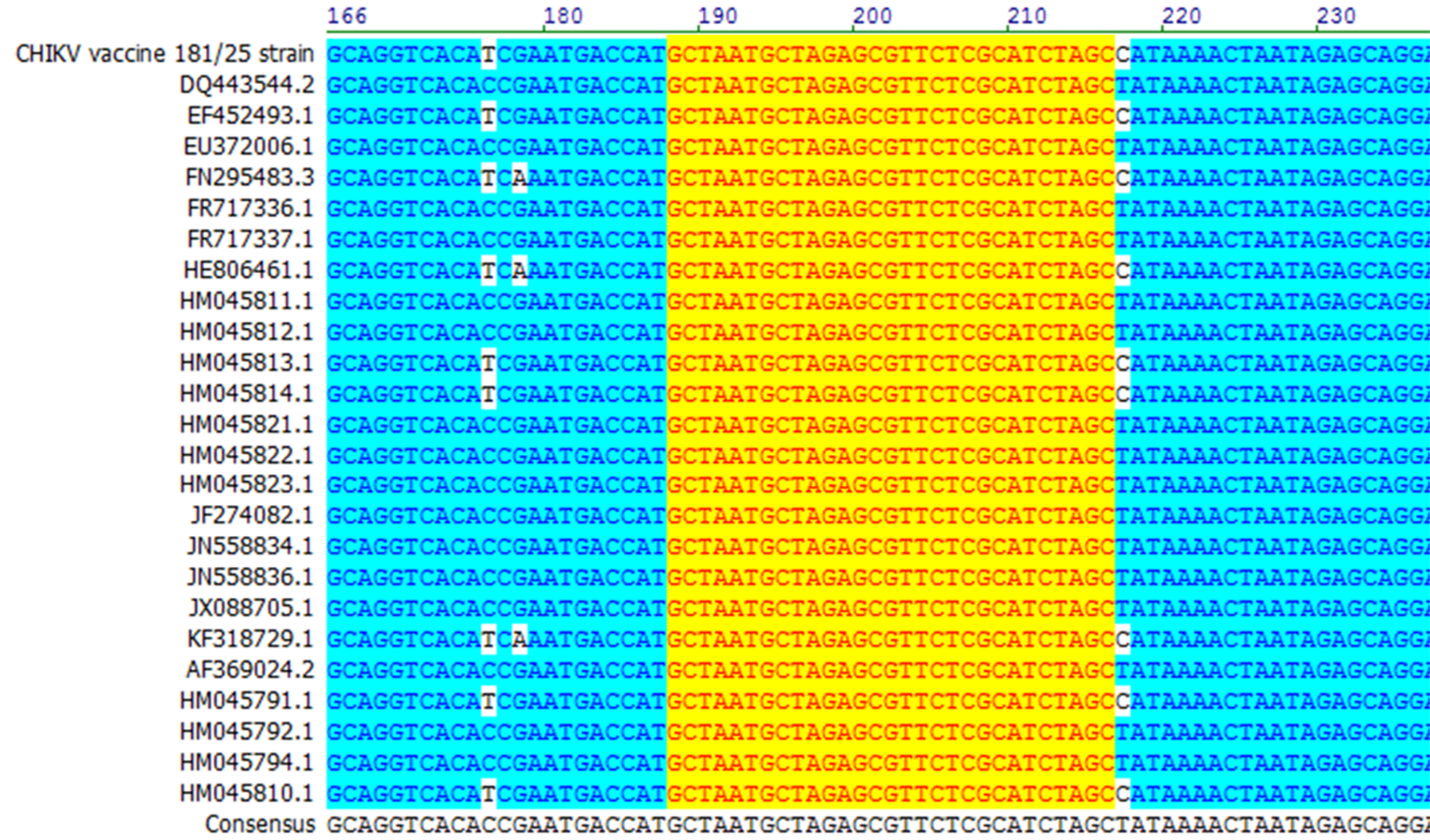

Supplement: S1 Fig — An alignment was performed of twenty five (25) CHIKV genomic sequences to determine the most optimal regions for the design of the chikungunya virus specific and DENV/CHIKV dual targeting antiviral group I introns by determining the region with the greatest conservation within the CHIKV RNA genomes. Nucleotide sequences in yellow indicate complete conservation. Nucleotide sequences in blue indicate partial conservation. Nucleotide sequence position is indicated at the top of the figure. GenBank Accession Numbers at the left of the figure indicate the CHIKV sequences aligned. (TIF) [file pone.0139899.s001.tif]

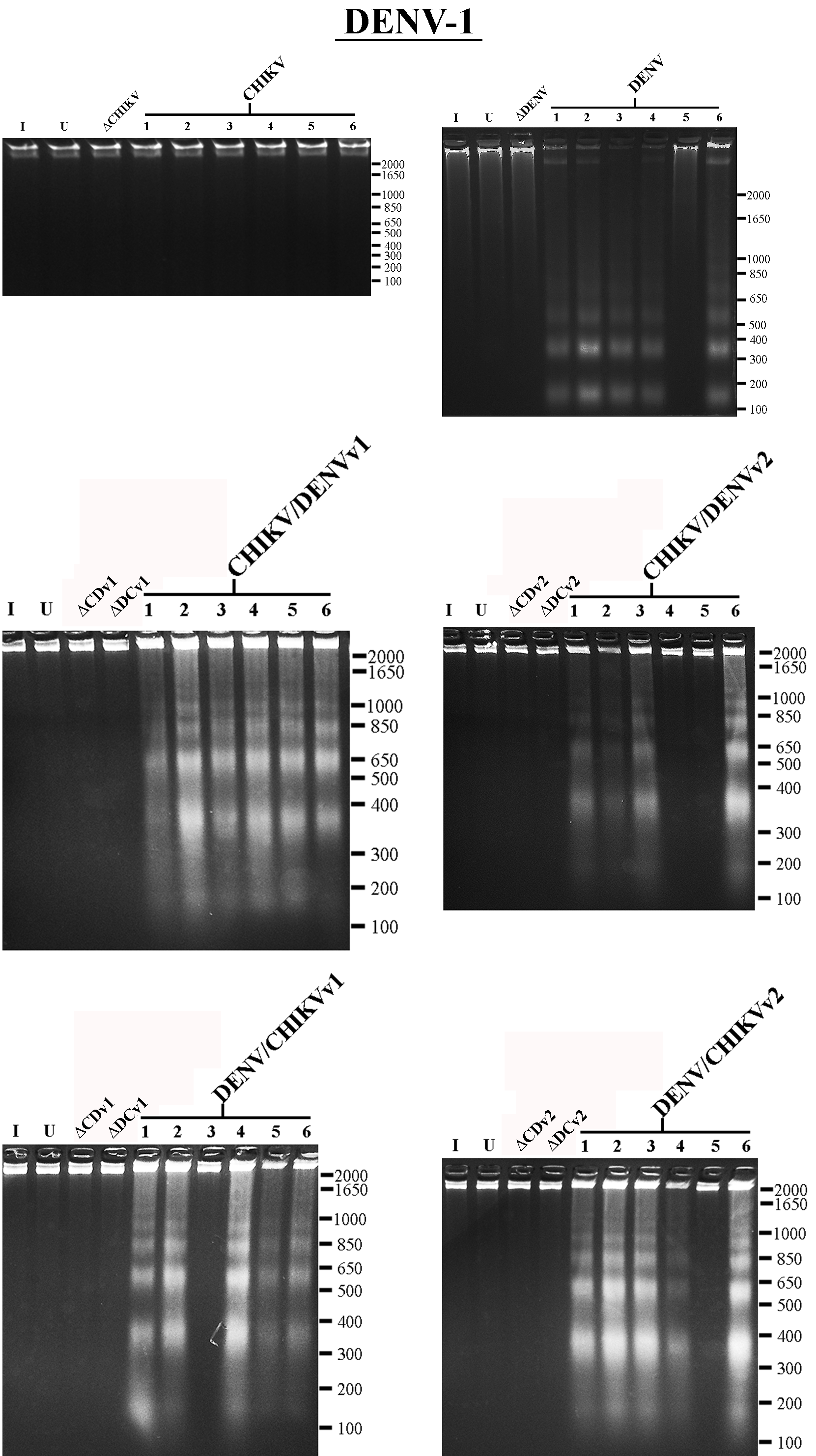

Supplement: S2 Fig — Clonal Ae. albopictus C6/36 cells transformed with the anti-CHIKV, anti-DENV or anti-CHIKV/DENV dual targeting antiviral intron constructs indicated were each challenged with dengue virus serotype 1, processed, and analyzed as described for Fig 7 and in Materials and Methods. I = Wt C6/36 mosquito cells infected with virus indicated; U = uninfected Wt C6/36 mosquito cells; ΔC/Dv1 = ΔCHIKV/DENVv1; ΔD/Cv1 = ΔDENV/CHIKVv1; ΔC/Dv2 = ΔCHIKV/DENVv2; ΔD/Cv2 = ΔDENV/CHIKVv2. (TIF) [file pone.0139899.s002.tif]

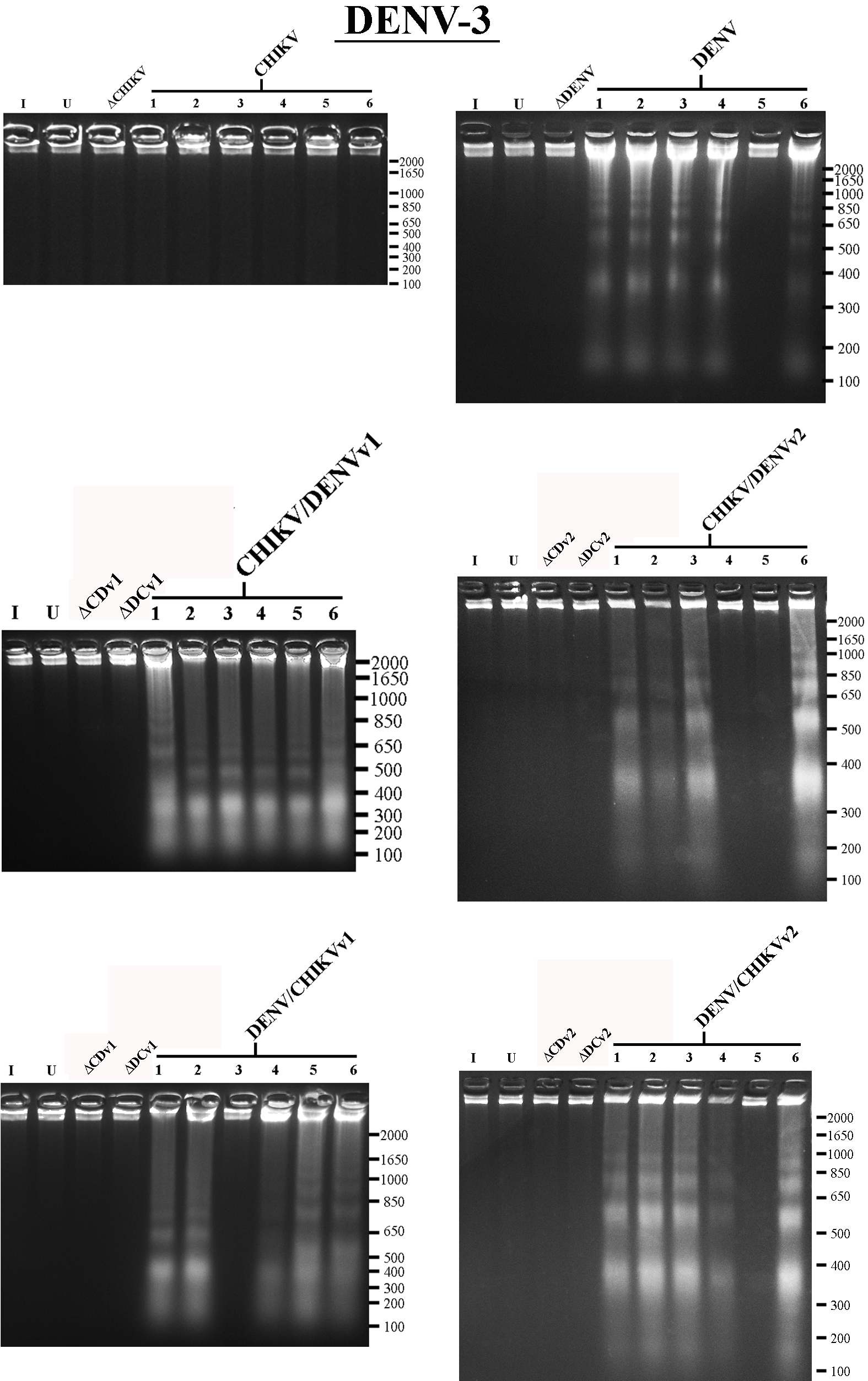

Supplement: S3 Fig — Clonal Ae. albopictus C6/36 cells transformed with the anti-CHIKV, anti-DENV or anti-CHIKV/DENV dual targeting antiviral intron constructs indicated were each challenged with dengue virus serotype 3, processed, and analyzed as described for Fig 7 and in Materials and Methods. I = Wt C6/36 mosquito cells infected with virus indicated; U = uninfected Wt C6/36 mosquito cells; ΔC/Dv1 = ΔCHIKV/DENVv1; ΔD/Cv1 = ΔDENV/CHIKVv1; ΔC/Dv2 = ΔCHIKV/DENVv2; ΔD/Cv2 = ΔDENV/CHIKVv2. (TIF) [file pone.0139899.s003.tif]

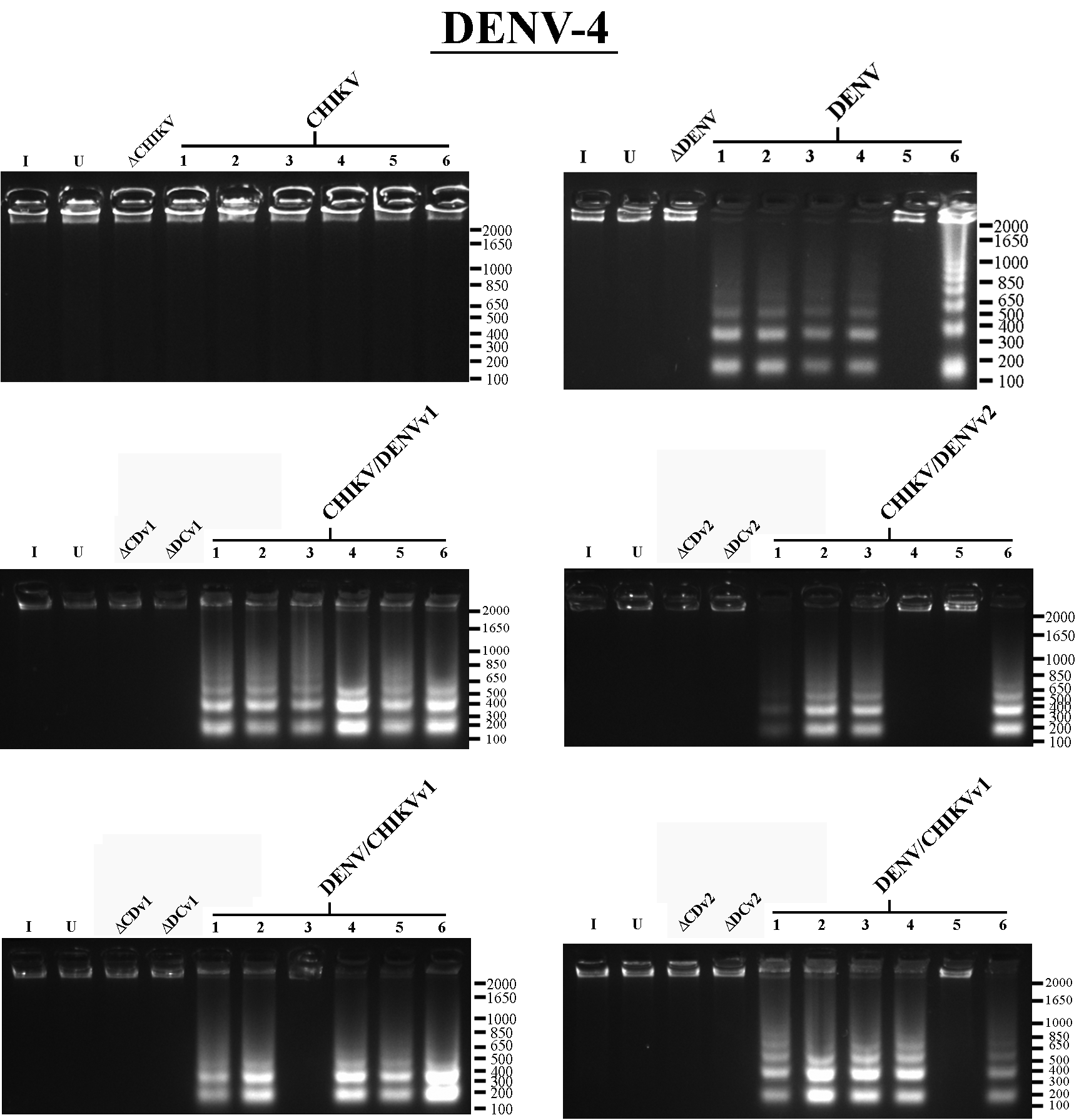

Supplement: S4 Fig — Clonal Ae. albopictus C6/36 cells transformed with the anti-CHIKV, anti-DENV or anti-CHIKV/DENV dual targeting antiviral intron constructs indicated were each challenged with dengue virus serotype 4, processed, and analyzed as described for Fig 7 and in Materials and Methods. I = Wt C6/36 mosquito cells infected with virus indicated; U = uninfected Wt C6/36 mosquito cells; ΔC/Dv1 = ΔCHIKV/DENVv1; ΔD/Cv1 = ΔDENV/CHIKVv1; ΔC/Dv2 = ΔCHIKV/DENVv2; ΔD/Cv2 = ΔDENV/CHIKVv2. (TIF) [file pone.0139899.s004.tif]

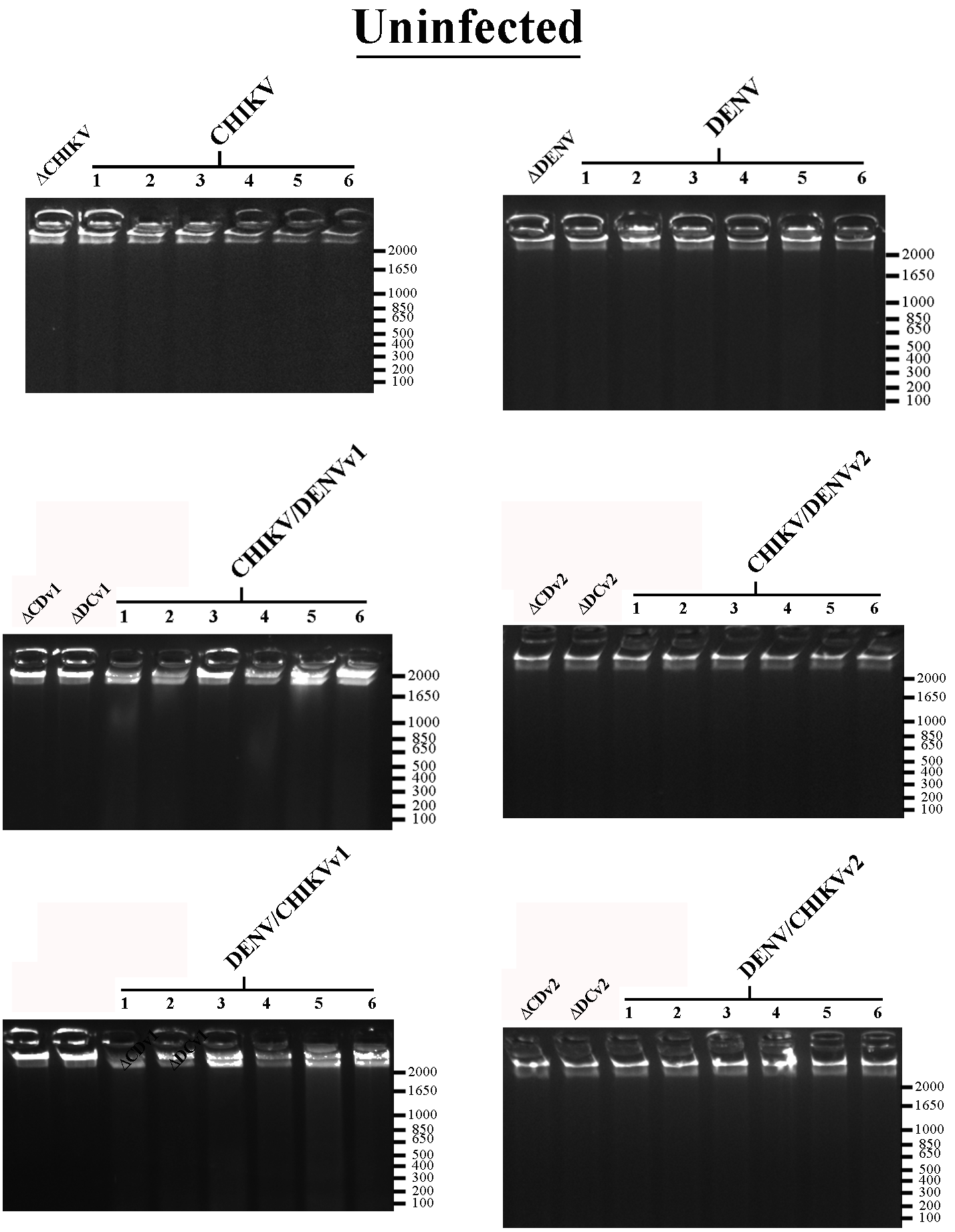

Supplement: S5 Fig — Clonal Ae. albopictus C6/36 cells transformed with the anti-CHIKV, anti-DENV or anti-CHIKV/DENV dual targeting antiviral intron constructs indicated were each mock infected, processed, and analyzed as described for Fig 7 and in Materials and Methods. I = Wt C6/36 mosquito cells infected with virus indicated; U = uninfected Wt C6/36 mosquito cells; ΔC/Dv1 = ΔCHIKV/DENVv1; ΔD/Cv1 = ΔDENV/CHIKVv1; ΔC/Dv2 = ΔCHIKV/DENVv2; ΔD/Cv2 = ΔDENV/CHIKVv2. (TIF) [file pone.0139899.s005.tif]

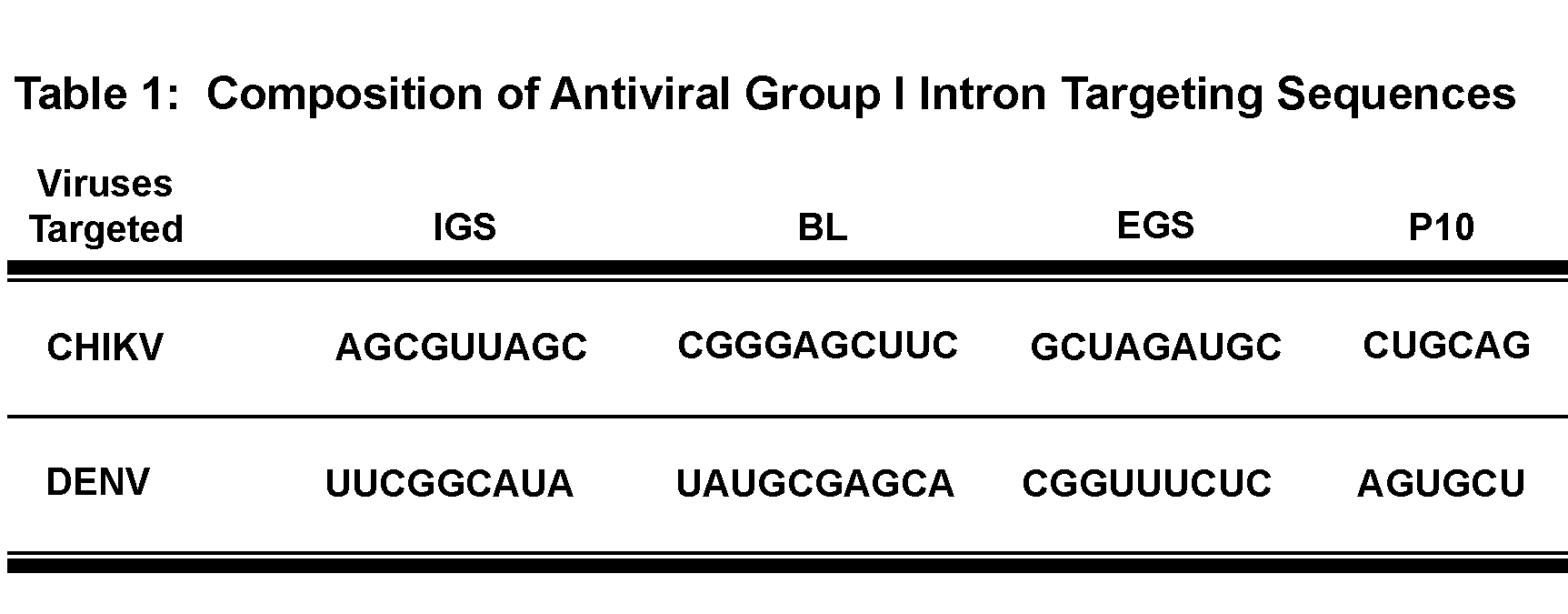

Supplement: S1 Table — The ribonucleotide sequences of each antiviral group I intron are shown. The left column lists the viruses targeted by the dual targeting introns containing respective targeting sequences shown. Targeting sequences specific for each virus are indicated. See methods for description of assembly. CHIKV = Chikungunya virus targeting sequences; DENV = Dengue virus targeting sequences; EGS = external guide sequence; IGS = internal guide sequence; BL = bulge loop; TSD = trans splicing domain; P10 = P10 helix. (TIF) [file pone.0139899.s006.tif]

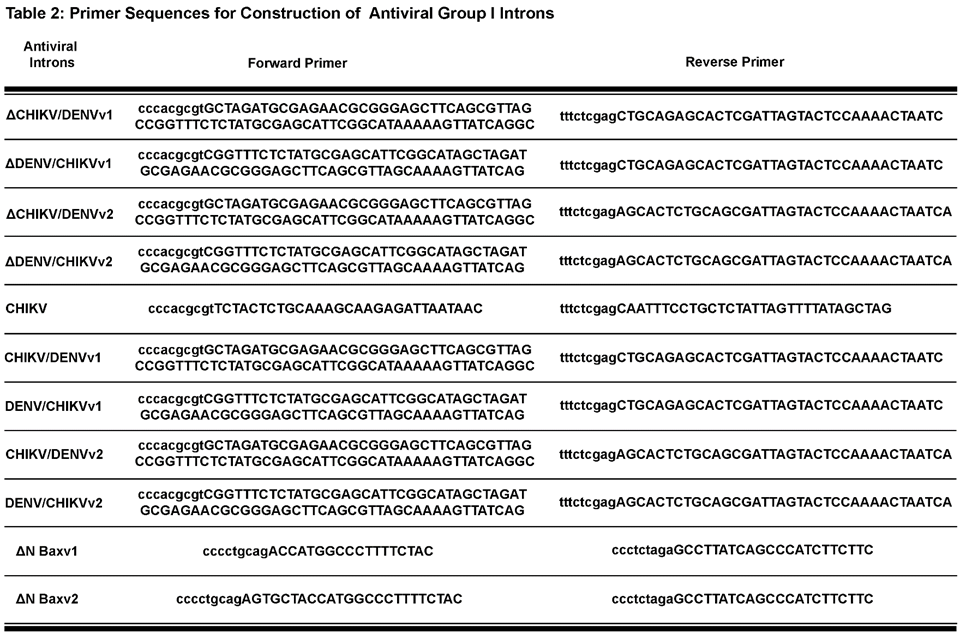

Supplement: S2 Table — Listed are the forward and reverse primer sets used to produce the PCR fragments of anti-CHIKV/DENV introns, negative controls, and the ΔN Bax 3’ exon for plasmid insertion. Restriction sites used are indicated by lowercase nucleic acids. See Materials and Methods for description of vector construct assembly. (TIF) [file pone.0139899.s007.tif]
